# The EphA2 receptor is activated through induction of distinct, ligand-dependent oligomeric structures

**DOI:** 10.1038/s42003-018-0017-7

**Published:** 2018-02-22

**Authors:** Deo R. Singh, Pranjali Kanvinde, Christopher King, Elena B. Pasquale, Kalina Hristova

**Affiliations:** 1 Department of Materials Science and Engineering, Johns Hopkins University, 3400 Charles Street, Baltimore, MD 21218 USA; 2 Institute of NanoBioTechnology, Johns Hopkins University, 3400 Charles Street, Baltimore, MD 21218 USA; 3 Program in Molecular Biophysics, Johns Hopkins University, 3400 Charles Street, Baltimore, MD 21218 USA; 4 Sanford Burnham Prebys Medical Discovery Institute, 10901 North Torrey Road, La Jolla, CA 92037 USA; 5 Pathology Department, University of California San Diego, La Jolla, CA 92093 USA

## Abstract

The EphA2 receptor tyrosine kinase is capable of activating multiple diverse signaling pathways with roles in processes such as tissue homeostasis and cancer. EphA2 is known to form activated oligomers in the presence of ephrin-A ligands. Here, we characterize the lateral interactions between full-length EphA2 molecules in the plasma membrane in the presence of three types of ligands (dimeric ephrinA1-Fc, monomeric ephrinA1, and an engineered peptide ligand) as well as in the absence of ligand, using a quantitative FRET technique. The data show that EphA2 forms higher-order oligomers and two different types of dimers that all lead to increased EphA2 tyrosine phosphorylation, which is indicative of increased kinase-dependent signaling. We find that different ligands stabilize conformationally distinct oligomers that are assembled through two different interfaces. Our results suggest that these different oligomeric assemblies could have distinct signaling properties, contributing to the diverse activities of the EphA2 receptor.

## Abstract (web summary)

Deo Singh et al. use Fully Quantified Spectral Imaging-FRET to show that the EphA2 receptor forms dimers or higher order oligomers depending on the type of ligand, and that different ligands stabilize EphA2 dimers through distinct interfaces. These findings may explain how EphA2 activates diverse signaling pathways.

## Introduction

The Eph receptors are the largest family of receptor tyrosine kinases and play critically important roles in tissue organization and homeostasis as well as in many pathological processes^1–3^. EphA2 has the strongest links to cancer of any of the 14 Eph receptors, and thus has been extensively studied^1, 4–6^. EphA2 is known to mediate diverse, and even opposite, effects through different signaling mechanisms^2, 7, 8^. The ligand and kinase activity-dependent form of EphA2 signaling involves receptor tyrosine phosphorylation and is potently induced by cell surface-anchored ephrinA ligands, such as ephrinA1, as well as by soluble forms of these ligands dimerized by fusion to Fc and clustered with anti-Fc antibodies^2^. Soluble monomeric forms of the ephrinA ligands, which can be released from cells by proteases, as well as engineered short peptide ligands can also promote EphA2 tyrosine phosphorylation and signaling through mechanisms that have remained mysterious^9–14^. EphA2 kinase-dependent signaling has been linked to a variety of functional outcomes, such as suppression of the AKT–mTORC1 and RAS–ERK oncogenic pathways and inhibition of cell adhesion and migration/invasion, but also enhancement of cancer cell dispersal and promotion of tumor angiogenesis^1–4, 8^. In addition, EphA2 signaling plays a role in inflammation, atherosclerosis, and infection^15^. EphA2 can also behave as an oncoprotein through another form of signaling that does not require either ligands or kinase activity and involves phosphorylation on S897 in the segment linking the kinase domain with the SAM domain^7^. EphA2 S897 phosphorylation is due to serine/threonine kinases such as AKT, RSK, and PKA and promotes cell migration/invasion, metastasis, and cancer stem cell-like features^1, 3, 7, 8, 16, 17^.

Given the high diversity of functional outcomes mediated by EphA2 kinase-dependent signaling, we asked whether this receptor may be capable of forming different types of oligomers (dimers or clusters) in the plasma membrane, depending on the nature of the activating ligand. To explore this possibility, we assessed the homo-association of EphA2 receptor molecules in the plasma membrane in the presence of three types of ligands (dimeric ephrinA1-Fc, monomeric ephrinA1, and an engineered peptide ligand) in comparison with EphA2 in the absence of ligand.

The architecture of the EphA2 receptor, like that of most other receptor tyrosine kinases, includes a large extracellular region, a single transmembrane helix, and an intracellular region containing a kinase domain. The extracellular region is composed of an N-terminal ligand-binding domain, a cysteine-rich domain, and two fibronectin type III domains. High-resolution structural information on the EphA2 extracellular region is available. In crystals, neighboring extracellular regions have been shown to interact with each other via two interfaces, often referred to as the “dimerization (or heterodimerization)” and “clustering” interfaces^18, 19^. We characterized the lateral interactions between full-length EphA2 molecules in the plasma membrane in quantitative terms, using a FRET-based spectral imaging methodology that reports the type and abundance of transmembrane protein oligomers^20, 21^. To probe the interfaces in the different EphA2 dimers and clusters, we used mutagenesis guided by the solved crystal structures of the EphA2 extracellular region^18, 19^. The distinctive effects we observed for mutations in the dimerization or the clustering interface show that EphA2 is capable of forming several oligomers that are stabilized through distinct interfaces.

## Results

### EphrinA1-Fc induces EphA2 clusters comprising two interfaces

Kinase-dependent signaling by the EphA2 receptor is strongly activated in cells stimulated with ephrinA1-Fc^22^. This ligand is a chimeric protein composed of ephrinA1 fused to the Fc region of an IgG_1_ antibody and binds to EphA2 with sub-nanomolar to low nanomolar affinity^23^. Ephrin Fc fusion proteins are dimeric and for some Eph receptors they have been shown to cause receptor clustering and high activation only when they are oligomerized with anti-Fc antibodies, mimicking the clustering induced by the endogenous plasma membrane-anchored ephrin ligands^2, 24–27^.

To determine whether dimeric ephrinA1-Fc induces EphA2 dimerization or higher order oligomerization, we performed FRET experiments in the presence of 5 μg per ml (~50 nM) ephrinA1-Fc. This concentration greatly exceeds the apparent dissociation constant, so that all EphA2 molecules are ligand-bound. We also sought to determine whether the interfaces between associated EphA2 extracellular domains in the full-length receptor in the plasma membrane of live cells correspond to the two interfaces predicted from the solved crystal structures of the EphA2 extracellular region^18, 19^. We further sought to quantify the fraction of EphA2 molecules that are in oligomers (dimers or clusters) as a function of receptor concentration, and to determine how perturbations in the two predicted interfaces affect the dimeric/clustered population of EphA2 molecules. We performed these investigations using the recently developed fully quantified spectral imaging-forster resonance energy transfer (FSI-FRET) method^20^. The EphA2 receptor was tagged at its C-terminus with the fluorescent proteins mTurquoise (mTurq, donor) or eYFP (acceptor) via a (GGS)_5_ flexible linker. These two fluorescent proteins are a FRET pair that has been previously successfully used in FSI-FRET experiments^20^, and we have previously shown that the attachment of the fluorescent proteins to the EphA2 C-terminus does not detectablyaffect phosphorylation of the receptor^28^.

The FSI method requires the acquision of complete FRET and acceptor spectra, and uses an assumption-free, fully resolved system of equations to calculate FRET efficiencies in the plasma membrane with high precision^20^. The method yields the FRET efficiency and the concentration of donor-labeled and acceptor-labeled receptors in the plasma membrane^20^. Determination of these two-dimensional concentrations, which also allows the generation of binding curves, is possible only if the cells have a flat, unwrinkled plasma membrane. This is achieved by subjecting the cells to hypo-osmotic conditions, which do not alter the FRET efficiencies measured for membrane proteins and thus their association^20^. It should be noted that hypo-osmotic conditions can occur physiologically, do not cause irreversible cell damage, and the changes they induce in cells are fully reversible^29–31^.

The data generated with the FSI-FRET method are interpreted within the context of thermodynamic models based on the Kinetic Theory of FRET^32^. Thermodynamic models are built for different types of oligomerization (with oligomer order = *n*) and fitted to the data to calculate the mean squared error (MSE). Extensive evaluation of this approach has shown that an MSE minimum at *n* = 2 reliably identifies dimer populations^21^. An MSE minimum at *n* > 2 or the same MSE value for different oligomer orders points to the presence of oligomers larger than dimers, although the presence of some dimers cannot be excluded^21^. While the exact order of the oligomers (*n *= 3, 4, 5, etc.) cannot be defined from this analysis, the method reliably yields the receptor fraction that exists in an oligomeric state (i.e., as dimers or clusters)^21^.

**Fig. 1:**
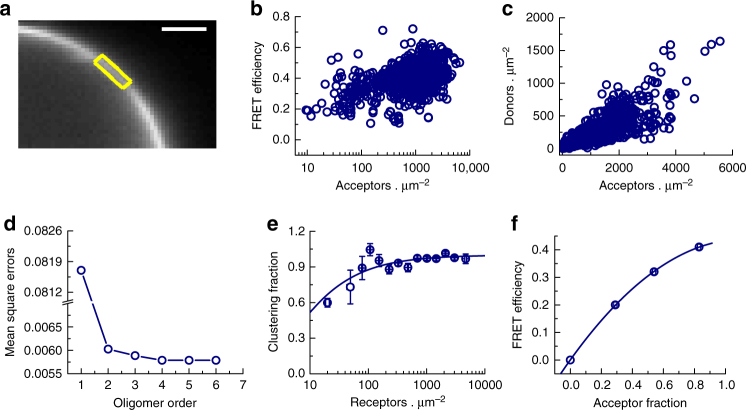
Dimeric ephrinA1-Fc induces EphA2 receptor clustering. **a** Portion of a HEK293T cell expressing EphA2-mTurq and EphA2-eYFP in hypo-osmotic medium, imaged when mTurq was excited. A plasma membrane region of homogeneous fluorescence, a few µm in length (yellow box), is analyzed to determine the EphA2-mTurq concentration, the EphA2-eYFP concentration and the FRET efficiency, as described in the Materials and methods. The scale bar is 5 μm. **b** FRET efficiency vs. acceptor (EphA2-eYFP) concentration. Each data point corresponds to one plasma membrane region. **c** Donor (EphA2-mTurq) concentration vs. acceptor (EphA2-eYFP) concentration in the selected membrane regions. In **b** and **c**, 275 cells were imaged in four independent experiments, yielding 858 data points. **d** Mean square error (MSE) vs. oligomer order. MSE is minimized for *n* > 4, indicating the presence of oligomers that are larger than dimers (i.e., clusters). **e** Clustered EphA2 receptor fraction as a function of total EphA2 concentration. The data were binned and the averages are shown along with the standard errors. The solid line represents the theoretical best fit to the data. **f** Mean FRET efficiencies vs. mean acceptor fractions, determined as shown in Supplementary Figure 1. The plot is based on more than 1000 data points. The dependence deviates from a linear function, supporting the conclusion that exposure to ephrinA1-Fc induces preferentially the formation of EphA2 clusters

We subjected HEK293T cells co-transfected with EphA2-mTurq and EphA2-eYFP to reversible hypo-osmotic conditions in the presence of 50 nM ephrinA1-Fc. The donor concentration, acceptor concentration and FRET efficiency were calculated for regions of ruffles-free membrane (Fig. 1a) using the FSI-FRET software^20^. The FRET efficiency vs. acceptor concentration (Fig. 1b) and the donor concentration vs. acceptor concentration (Fig. 1c) were used to calculate the mean MSE as a function of the oligomer order *n*, showing a minimum value for *n* ≥ 4 (Fig. 1d**)** and thus indicating that EphA2 forms oligomers larger than dimers (i.e., clusters) in the presence of ephrinA1-Fc. The fraction of clustered EphA2 calculated from the data exceeds 70% over the entire receptor concentration range, demonstrating the effectiveness of ephrinA1-Fc in clustering EphA2 even when the receptor is present at low concentrations in the plasma membrane (Fig. 1e). For concentrations of 600 receptors per μm^2^, the EphA2 expression estimated for A549 lung cancer cells^33^, 100% of the EphA2 molecules are in clusters.

The dependence of the apparent FRET efficiency on acceptor concentration is used in the literature to differentiate between dimers and higher order oligomers^34–36^. The FRET efficiency was measured in the presence of 50 nM ephrinA1-Fc and different donor to acceptor ratios (3:1, 1:1, 1:3; Supplementary Figure 1a-f). The average FRET efficiency values and acceptor fractions for different donor to acceptor ratios were determined from the histograms shown in Supplementary Figure 1g, h. Only data for total EphA2 concentrations that exceeded 100 receptors per μm^2^ were included, since at these concentrations the oligomeric fraction exceeds 90% and FRET depends mainly on the acceptor fraction but only weakly on the total receptor concentration. The plot of average FRET efficiency vs. average acceptor fraction is non-linear (Fig. 1f), as expected for cluster formation. Thus, this method confirms that dimeric ephrinA1-Fc induces the formation of EphA2 clusters.

**Fig. 2:**
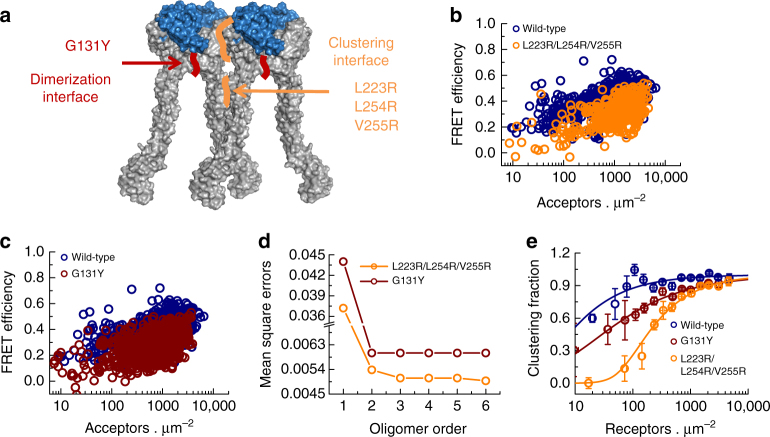
Interfaces involved in EphA2 receptor clustering induced by ephrinA1-Fc. **a** Crystal structure showing a lateral view of four EphA2 extracellular regions (gray) bound to four ephrinA1 molecules (light blue; PDB ID: 3MX0). The receptor tetramer is stabilized via two interfaces: the “clustering” interface (approximately outlined in orange), which includes contacts mediated by L223, L254, and V255 in the cysteine-rich domain, and the dimerization interface (approximately outlined in wine), which includes contacts mediated by G131 in the ligand-binding domain^18^. **b** Comparison of raw FRET data for EphA2 wild-type and the L223R/L254R/V255R mutant in the presence of ephrinA1-Fc. In this experiment, 275 cells were imaged in four independent experiments to obtain 858 data points for the wild-type, and 196 cells were imaged in four independent experiments to obtain 563 data points for the L223R/L254R/V255R mutant. **c** Comparison of raw FRET data for EphA2 wild-type and the G131Y mutant in the presence of ephrinA1-Fc. A total of 618 cells were imaged in six independent experiments to yield 2310 data points for the G131Y mutant. **d** MSE vs. oligomer order for the L223R/L254R/V255R and G131Y mutants in the presence of ephrinA1-Fc. The MSE minimum for the L223R/L254R/V255R mutant occurs at *n* = 6. The MSE for the G131Y mutant is the same for *n* ≥ 2. As previously shown^21^, these results indicate that the EphA2 receptor is preferentially assembled into clusters, although the presence of some dimers cannot be excluded. **e** Representation of the clustered fractions for EphA2 wild-type and the L223R/L254R/V255R and G131Y mutants as a function of total receptor concentration shows that mutation of both interfaces decreases the fraction of clustered EphA2

Clusters of EphA2 extracellular regions in crystals are stabilized via two distinct interfaces (Fig. 2a). One of these interfaces does not involve the bound ephrin ligand and is referred to as the “clustering interface”^18^. Contacts within this clustering interface involve residues L223, L254, and V255 in the cysteine-rich domain. The second interface (termed the “dimerization interface”, or the “heterodimerization interface” when the ligand is also considered) is stabilized by receptor–ligand and receptor–receptor contacts in the ligand-binding domain, including contacts involving amino acid G131. We therefore used an EphA2 L223R/L254R/V255R triple mutant^18, 37^ and an EphA2 G131Y mutant to separately destabilize each of the two interfaces. The raw FRET data for EphA2 wild-type and the two mutants in the presence of ephrinA1-Fc are compared in Fig. 2b, c. MSE analysis of the FRET data shows that both mutants also form clusters (Fig. 2d; see also Supplementary Figure 2 for L223R/L254R/V255R mutant data confirming clustering). The fraction of mutant EphA2 in clusters is reduced, however, demonstrating that both sets of mutations destabilize the clusters (Fig. 2e). These data are consistent with the involvement of both interfaces in EphA2 clustering induced by ephrinA1-Fc. The G131Y mutation appears to have a weaker effect, likely due to a contribution of the bound ephrin ligand, or simply because only a single-amino acid is mutated.

**Fig. 3:**
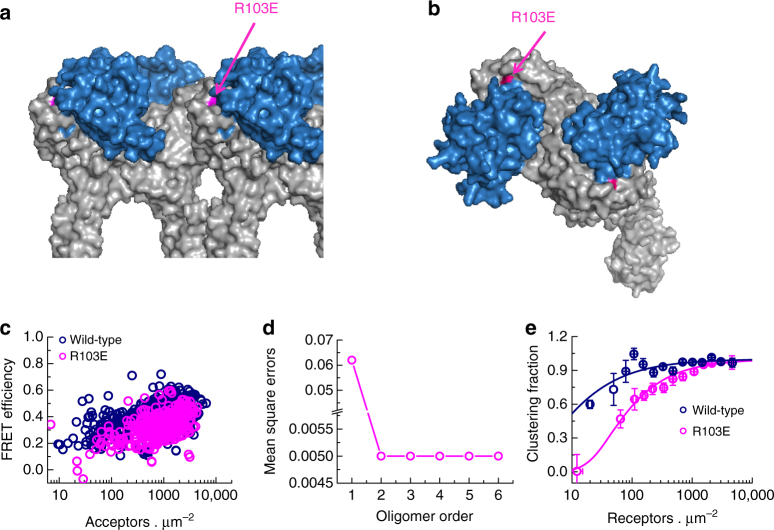
Effect of the R103E mutation on EphA2 clustering induced by ephrinA1-Fc. **a**, **b** Side and top views of a crystal structure of four EphA2 molecules (gray) bound to four ephrinA1 molecules (light blue; PDB ID: 3MX0). The position of the R103E mutation in two of the EphA2 molecules is shown in magenta, and indicated by an arrow in one. **c** Comparison of raw FRET data for EphA2 wild-type and the R103E mutant in the presence of ephrinA1-Fc. 275 cells were imaged in four independent experiments to obtain 858 data points for the wild type. A total of 201 cells were imaged in three independent experiments to obtain 474 data points for R03E mutant. **d** MSE vs. oligomer order for EphA2 R103E in the presence of ephrinA1-Fc. The MSE value is the same for all *n* ≥ 2, indicating oligomerization with predominance of clusters. **e** Comparison of EphA2 wild-type and R103E mutant clustered fractions in the presence of saturating concentration of ephrinA1-Fc shows that the R103E mutation severely destabilizes the clusters

Crystallographic studies have shown that R103 in the ephrin-binding pocket of EphA2 (Fig. 3a, b) forms a salt bridge with the ephrinA1 ligand^38^. This is a critical contact for ephrin binding because activation of EphA2 by ephrinA1-Fc is severely impaired by the R103E mutation^38^. FRET experiments show that the EphA2 R103E mutant can form clusters in the presence of ephrinA1-Fc (Fig. 3d; Supplementary Figure 3), but these clusters have reduced stability compared to EphA2 wild-type clusters (Fig. 3e, see also raw FRET data in Fig. 3c). Thus, the reduction in ephrinA1-Fc binding affinity due to the R103E mutation correlates with a reduction in the fraction of clustered receptor.

### Unliganded EphA2 forms dimers via the clustering interface

**Fig. 4:**
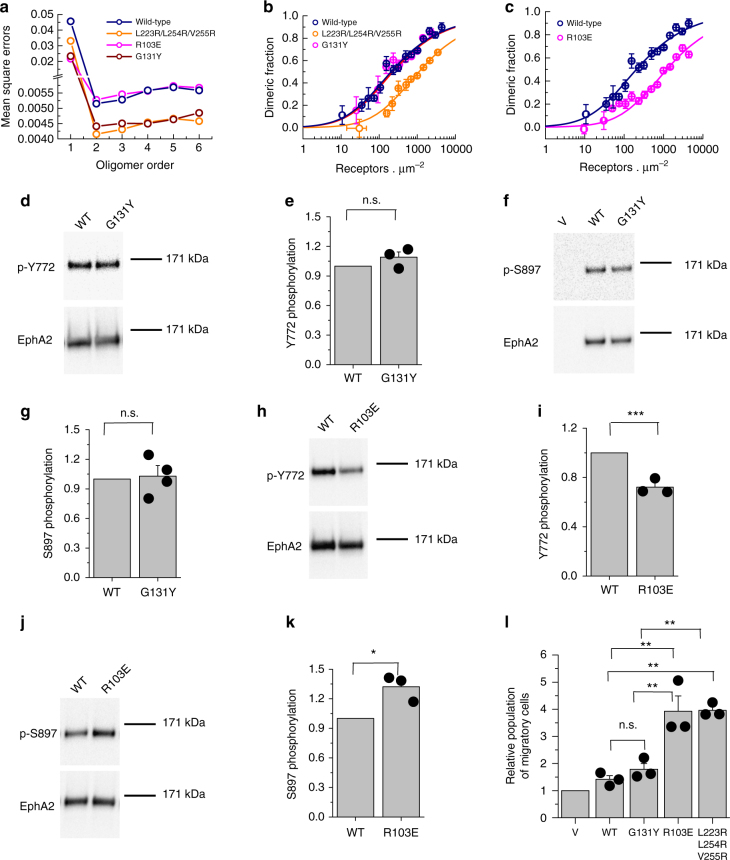
Dimerization of EphA2 wild-type and the L223R/L254R/V255R, G131Y and R103E mutants in the absence of ligand binding. **a** MSE vs. oligomer order for EphA2 wild-type and the three mutants. MSE is minimized at *n* = 2 for all, indicating the presence of dimers. **b** Dimerization curves for EphA2 wild-type and the L223R/L254R/V255R and G131E mutants. The L223R/L254R/V255R mutations reduce dimerization, while the G131Y mutation has no effect. Thus, the unliganded dimer is stabilized through the “clustering interface”. **c** Dimerization curves for EphA2 wild-type and the R103E mutant. The R103E mutant exhibits a reduced dimerization propensity, despite the fact that this residue is not part of the clustering interface. **d** A representative Western blot comparing Y772 phosphorylation for EphA2 wild-type and the G131Y mutant. **e** Quantification from three independent experiments (shown as solid circles) shows no statistically significant difference (*p* > 0.05 from Student’s *t*-test). **f** A representative Western blot image comparing S897 phosphorylation for EphA2 wild-type and the G131Y mutant. **g** Quantification from four independent experiments (shown as solid circles) shows no statistically significant difference (*p* > 0.05 from Student’s *t*-test). **h** Representative Western blot images comparing Y772 phosphorylation for EphA2 wild-type and the R103E mutant. **i** Quantification from three independent experiments (shown as solid circles) shows that the R103E mutant has lower Y772 phosphorylation than EphA2 wild-type (***p < 0.001 from Student’s *t*-test). **j** A representative Western blot comparing S897 phosphorylation for EphA2 wild-type and the R103E mutant. **k** Quantification from three independent experiments (shown as solid circles) shows that the R103E mutant has higher S897 phosphorylation than EphA2 wild-type (*p < 0.05 from Student’s *t*-test). **l** Cell migration assays with HEK293T cells expressing EphA2 wild-type and the three mutants. The solid circles represent the the individual experiments. Cells expressing EphA2 wild-type and the G131Y mutant exhibit similar migratory ability. In contrast, cells expressing the L223R/L254R/V255R mutant and the R103E mutant migrate faster than wild-type. (**p < 0.01 from ANOVA, n.s. non-significant, *p* > 0.05). The bars in e, g, i and k represent the averages from different experiments with the standard errors

We have previously shown that EphA2 can form dimers even in the absence of ligand binding, and that these unliganded dimers are destabilized by the L223, L254 and V255 set of mutations in the clustering interface^37^. To determine whether EphA2 unliganded dimers are also affected by the G131Y mutation in the dimerization interface, we compared EphA2 wild-type and the L223R/L254R/V255R and G131Y mutants in the absence of treatment with a ligand. MSE analysis of the FRET data shows that EphA2 wild-type and the two mutants all form predominantly dimers without a significant higher order oligomer population (Fig. 4a; see Supplementary Figures 4a-f for raw data). The stability of the EphA2 G131Y unliganded dimers is the same as for EphA2 wild-type, while the L223R/L254R/V255R mutations decrease dimer stability (Fig. 4b; Table 1 and Supplementary Table 1). Thus, G131 and the dimerization interface likely do not play a role in unliganded dimerization, which is mediated exclusively by the clustering interface.

**Table 1:** Dissociation constants for EphA2 wild-type and mutant dimers

EphA2 construct	No ligand *K*_diss_ (receptors per µm^2^)	m-ephrinA1 *K*_diss_ (receptors per µm^2^)	YSA peptide *K*_diss_ (receptors per µm^2^)
Wild-type	206 (133 to 278)	11 (5 to 18)	69 (46 to 92)
L223R/L254R/V255R	**1100 (628 to 1582)**	14 (2 to 25)	**428 (325 to 530)**
G131Y	230 (122 to 338)	**48 (32 to 64)**	71 (41 to 101)
R103E	**1200 (851 to 1564)**	**500 (320 to 680)**	**319 (206 to 432)**

*K*_diss_ is the dissociation constant (receptors per µm^2^) measured for EphA2 wild-type and mutant dimers and the uncertainties (in parentheses) are the 95% confidence intervals determined from the least square fit. In bold are the significantly increased dissociation constants compared to EphA2 wild-type, indicating significantly reduced dimer stability. In all these cases, *p* < 0.0001, based on Welch’s *t*-test with Bonferroni correction

Since the R103E mutation is expected to only affect ligand binding, it should not play a role in unliganded EphA2 dimerization. We found that the EphA2 R103E mutant forms dimers in the absence of ligand binding, but these dimers have decreased stability (Fig. 4c; Table 1 and Supplementary Table 1; see Supplementary Figure 4g, h for raw data). This result suggests allosteric effects, because R103 is part of the ephrin-binding pocket and not the clustering interface (Fig. 3a, b).

A correlation exists between EphA2 dimerization propensity, tyrosine phosphorylation and phosphorylation on S897 in transiently transfected HEK293T cells. We have previously shown that the L223R/L254R/V255R mutations decrease Y772 phosphorylation and increase S897 phoshorylation in the absence of ligand binding^37^. Here we show that the R103E mutation has similar effects, whereas the G131Y mutation does not affect unliganded EphA2 phosphorylation (Fig. 4d-k), in accordance with the FRET dimerization data.

We have also previously shown that a decrease in EphA2 dimerization correlates with an increase in the migration of EphA2-expressing HEK293T cells^37^. We observed enhanced migration in cells expressing the EphA2 L223R/L254R/V255R and R103E mutants compared to cells expressing EphA2 wild-type (Fig. 4l). In contrast, the G131Y mutation had no detectable effect on cell migration. These data are consistent with the observation that the L223R/L254R/V255R and R103E mutations, but not the G131Y mutation, reduce the propensity of EphA2 to form dimers in the absence of ligand binding and affect receptor phosphorylation.

### m-ephrinA1 dimerizes EphA2 via the dimerization interface

The ephrinA1 ligand is a cell surface-anchored protein that can undergo clustering in the plasma membrane^5^. However, ephrinA1 can also be released from cancer cells by proteolytic cleavage and can activate EphA2 as a soluble monomeric protein, although the mechanism is unknown^9, 12^. We asked whether monomeric ephrinA1 (m-ephrinA1) affects EphA2 lateral association and sought to characterize the nature of the association. Experiments were performed in the presence of 5 μg per ml (~200 nM) m-ephrinA1, a concentration that exceeds the dissociation constant of about 20–30 nM^10^. Thus, most of the EphA2 receptors are expected to be ligand-bound.

**Fig. 5:**
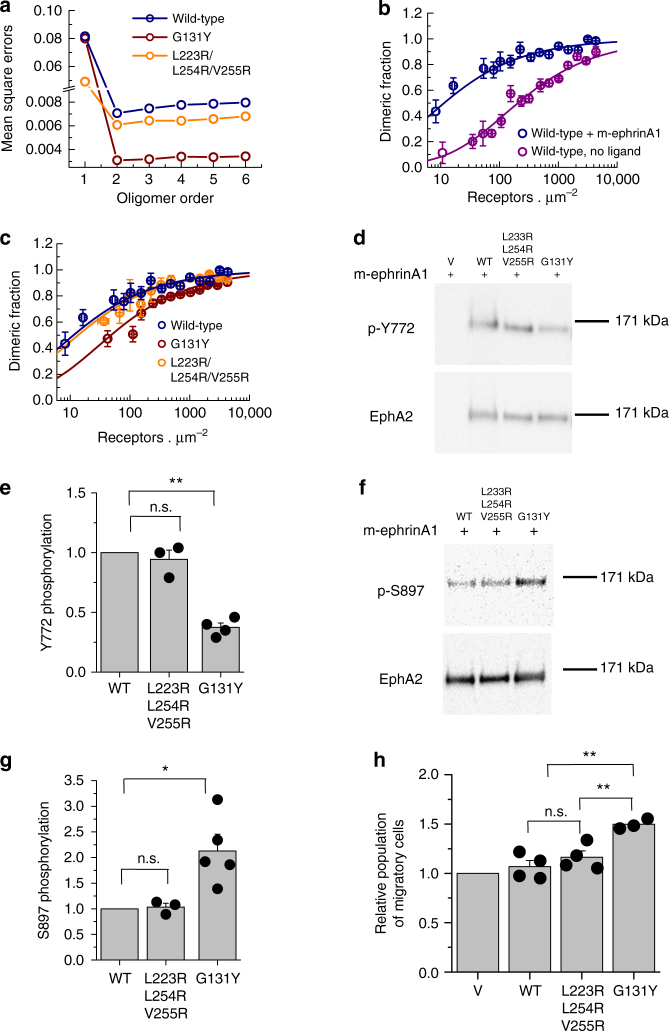
EphA2 dimerization induced by the monomeric ephrinA1 ligand. **a** MSE vs. oligomer order for EphA2 wild-type and the L223R/L254R/V255R and G131Y mutants in the presence of 200 nM m-ephrinA1. The MSEs are all minimized for *n* = 2, indicating dimerization. **b** Comparison of EphA2 dimerization propensity in the presence and absence of m-ephrinA1 shows that m-ephrinA1 significantly enhances EphA2 dimerization. **c** Dimerization curves in the presence of m-ephrinA1 show that the dimerization propensity of EphA2 wild-type and the L223R/L254R/V255R mutant are the same, while the G131Y mutant has a reduced dimerization propensity, indicating the involvement of the dimerization interface. **d** A representative Western blot showing Y772 phosphorylation of EphA2 wild-type and the indicated mutants following a 15 min stimulation with m-ephrinA1 and FBS. **e** Quantification of Y772 phosphorylation from three to four independent measurements is shown as solid circles. The bars represent the averages and the standard errors. EphA2 wild-type and the L223R/L254R/V255R mutant exhibit similar levels of Y772 phosphorylation while the G131Y mutant shows significantly lower phosphorylation (**p < 0.01 from Student *t*-test with Bonferroni correction). **f** Representative Western blots showing the S897 phosphorylation of EphA2 wild-type and mutants following a 15 min stimulation with m-ephrinA1 and FBS. **g** Quantification of S897 phosphorylation from three to five independent measurements is shown as solid circles. The bars represent the averages with standard errors. The wild-type and the L223R/L254R/V255R mutant show similar S897 phosphorylation while the G131Y mutant shows significantly higher phosphorylation (*p < 0.02 from Student *t*-test with Bonferroni correction). **h** Migration of HEK293T cells expressing wild-type and mutant EphA2 in the presence of m-ephrinA1. The solid circles represent the data points from three to four independent measurements. The bars represent the averages with standard errors. Cells expressing EphA2 wild-type and the L223R/L254R/V255R mutant exibit similar migratory propensity while cells expressing the G131Y EphA2 mutant migrate faster. (**p < 0.01 from ANOVA)

The FRET experiments revealed that EphA2 wild-type, the L223R/L254R/V255R mutant, and the G131Y mutant all form dimers in the plasma membrane when bound to m-ephrinA1 (Fig. 5a; Supplementary Figures 5 and 6). As would be expected, the m-ephrinA1-bound dimers are more stable than the unliganded dimers (Fig. 5b; Supplementary Figures 7, 8 and 9; Table 1 and Supplementary Table 2). Interestingly, the L223R/L254R/V255R set of mutations does not affect the stability of the EphA2 dimer bound to m-ephrinA1, whereas the G131Y mutation decreases dimer stability (Fig. 5c and Table 1). Thus, the m-ephrinA1-bound EphA2 dimers are stabilized through interactions within the dimerization interface and not the clustering interface, which stabilizes the unliganded dimers. This supports the notion that unliganded and m-ephrinA1-bound dimers have different configurations.

Consistent with the FRET data, we found that in the presence of m-ephrinA1 the G131Y mutant has lower Y772 phosphorylation and higher S897 phosphorylation compared to EphA2 wild-type, whereas the L223R/L254R/V255R mutant exhibits similar Y772 and S897 phosphorylation as EphA2 wild-type (Fig. 5d-g). Furthermore, cells expressing the G131Y mutant migrate faster in the presence of m-ephrinA1 compared to cells expressing EphA2 wild-type, whereas cells expressing the L223R/L254R/V255R mutant show the same migratory behavior as cells expressing EphA2 wild-type (Fig. 5h). These findings are consistent with the correlation between EphA2 dimerization, phosphorylation and cell migration reported earlier^28–30^.

**Fig. 6:**
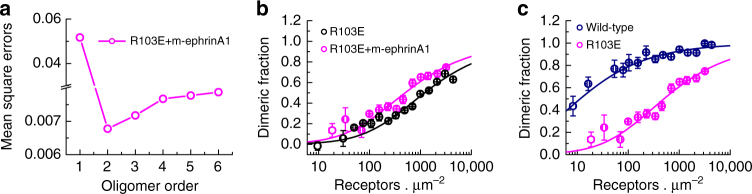
Effect of the R103E mutation on EphA2 dimerization in the presence of monomeric ephrinA1 ligand. **a** MSE vs. oligomer order for the EphA2 R103E mutant in the presence of m-ephrinA1. The MSE is miminized for *n* = 2, indicating dimerization. **b** Dimerization propensity of the EphA2 R103E mutant in the absence and in the presence of m-ephrinA1. The ligand slightly enhances dimerization, indicating that the EphA2 R103E mutation does not completely abrogate m-ephrinA1 binding. **c** Dimerization curves for EphA2 wild-type and the R103E mutant in the presence of m-ephrinA1. The R103E mutation decreases the stability of the dimers, most likely because it severely impairs m-ephrinA1 binding

FRET experiments revealed that the EphA2 R103E mutant in the presence of m-ephrinA1 is also a dimer (Fig. 6a; Supplementary Figure 10), which is only slightly more stable than the unliganded dimer (Fig. 6b) and drastically less stable than the EphA2 wild-type dimer bound to m-ephrinA1 (Fig. 6c). These differences are likely due to a strong decrease in ligand-binding affinity caused by the R103E mutation^38^. Thus, decreased ligand binding and decreased dimerization are likely responsible for the impaired biological effects reported for the EphA2 R103E mutant^38^.

### The YSA peptide dimerizes EphA2 via the clustering interface

**Fig. 7:**
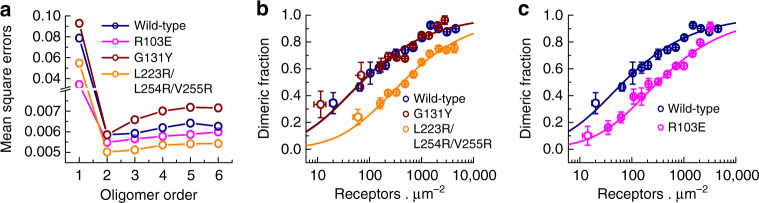
EphA2 dimerization in the presence of the monomeric YSA peptide ligand. **a** MSE vs. oligomer order for EphA2 wild-type and the L223R/L254R/V255R, G131Y, and R103E mutants. In all cases, the MSE minimum occurs at *n* = 2, indicating dimerization. **b** Dimerization curves show that the dimerization propensity of EphA2 wild-type and the G131Y mutant are the same, while the L223R/L254R/V255R mutant has reduced dimerization propensity. Data for the wild-type and the L223R/L254R/V255R mutant are from ref. ^30^. **c** Dimerization curves for EphA2 wild-type and the R103E mutant show that the mutant has reduced dimerization propensity

We previously showed that a short peptide (YSAYPDSVPMMSGSGSK) binds to the ephrin-binding pocket of EphA2 with a *K*_D_ of ~200 nM and a 1:1 binding stoichiometry^11, 13, 39–41^. Despite being monomeric, this “YSA” peptide is an agonist that promotes EphA2 tyrosine phosphorylation and activation. We performed FRET experiments in the presence of 6 μM YSA peptide in the culture medium, thus ensuring that most or all of the EphA2 molecules are bound to YSA. We have previously shown that the YSA peptide ligand under these conditions stabilizes EphA2 wild-type and L223R/L254R/V255R dimers^30^. Here we show that the EphA2 G131Y and R103E mutants also form dimers that are stabilized by YSA (Fig. 7a; Supplementary Figures 11 and 12; Table 1 and Supplementary Table 3). Comparison of the dimerization curves shows that in the presence of YSA, the L223R/L254R/V255R mutant exhibits lower dimerization propensity compared to EphA2 wild-type^30^, while the EphA2 G131Y mutant exhibits similar dimerization propensity as EphA2 wild-type (Fig. 7b and Table 1). The R103E mutation causes significant decrease in dimer stability compared to the wild-type receptor (Welch’s *t*-test, *p* < 0.0001; Fig. 7c and Table 1). This is likely the result of decreased YSA-binding affinity^13^ perhaps also combined with an allosteric mechanism, as hypothesized for EphA2 unliganded dimers. Overall, these results suggest that the YSA-bound EphA2 dimer is stabilized by the same contacts that stabilize the unliganded dimer, involving residues L233, L254, and V255 in the EphA2 clustering interface and not G131 in the dimerization interface. Thus, the binding of two different monomeric ligands, m-ephrinA1 and the YSA peptide, induces structurally distinct dimers.

## Discussion

EphA2 signaling is complex and can have diverse effects on cell behavior^1, 2, 7, 8^. For example, EphA2 has been implicated in both decreased and increased cell migration, and has been shown to both inhibit and promote cancer cell malignancy. Intriguingly, EphA2 kinase-dependent signaling has been linked to opposite effects on the activity of some downstream signaling proteins^3^, but the molecular mechanisms underpinning these opposite effects are currently unknown. While the cellular context may contribute to differences in EphA2 signaling activities, the biophysical characterization of EphA2 assemblies presented here could also provide a mechanistic explanation for diverse biological responses, as it reveals that different ligands can stabilize conformationally diverse oligomers that could have distinctive signaling properties.

**Fig. 8:**
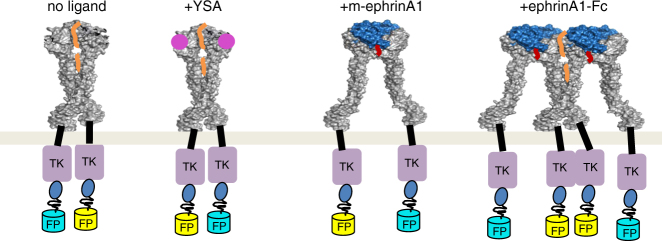
Cartoon representation of the findings: EphA2 can associate into two types of dimers as well as clusters, depending of the nature of the activating ligand. TK tyrosine kinase, FP fluorescent protein

We have used quantitative FRET to characterize the oligomerization of full-length EphA2 in the plasma membrane of HEK293T cells, in the absence of ligand and in the presence of different ligands, including dimeric ephrinA1-Fc, monomeric m-ephrinA1, or an engineered short peptide that targets the ligand-binding site of EphA2. By mutagenizing amino acids that contribute to two previously proposed interaction surfaces of EphA2^18, 19^, the dimerization and the clustering interface, we have gained insight into the architecture of EphA2 oligomeric assemblies in cells. We show that in the absence of ligand-binding EphA2 can form dimers that are stabilized through the clustering, but not the dimerization interface. Binding of the YSA peptide ligand, which appears to be monomeric, further stabilizes the same type of dimer, through interactions within the clustering interface. In contrast, the action of the monomeric ligand m-ephrinA1 is distinctly different, as it leads to the formation of an alternate dimer that is stabilized through the dimerization interface (Fig. 8). Crystallographic studies suggest that this receptor–receptor interface is complemented by the interface formed by the bound ephrinA1 molecules^18^. Finally, the binding of the dimeric ephrinA1-Fc ligand leads to the formation of larger oligomers that are stabilized via both interfaces (Fig. 8). Thus, our biophysical FSI-FRET data reveal that EphA2 can associate into clusters and at least two different types of dimers in response to different ligands. It is conceivable that the different EphA2 oligomers induced by different ligands can cause specific biological outcomes. This is perhaps analogous to the “biased agonism” of G protein-coupled receptors, which leads to functional selectivity and activation of different signaling pathways through the same receptor^42–44^. We therefore put forward the hypothesis that some of the EphA2 diverse actions may be due to different physical-chemical interactions within Eph receptor assemblies induced by different ligands.

As compared to other receptor tyrosine kinases, EphA2 signaling is much more complex as it involves cluster formation^2^. A second difference with most receptor tyrosine kinases lays in the action of the receptor monomer. While the monomers of other receptor tyrosine kinases are inactive, EphA2 monomers can be phosphorylated on S897 to promote pro-oncogenic activities such as cell migration and metastatic ability^7, 16, 17^. Therefore, the EphA2 monomers, along with the dimers and the clusters, represent distinct EphA2 signaling entities. It is worth noting that for moderate to high EphA2 expression levels such as 600 receptors per μm^2^ (see ref. ^33^), a substantial portion of the receptors is monomeric at saturating m-ephrinA1 and YSA concentrations. Thus, EphA2 monomers can co-exist with the dimers on the cell surface, and mediate different biological functions.

Our FRET experiments yield several unexpected findings. First, they suggest that the binding of m-ephrinA1 to EphA2 may promote a conformational switch that favors the alternative dimerization interface, which also includes receptor–ephrin contacts. Such an effect could not be predicted based on the crystal structure of the isolated EphA2 extracellular region solved in the presence of a monomeric ephrinA ligand, since EphA2 extracellular regions form clusters engaging both interfaces under the conditions used for crystallization. It is interesting that in our experiments m-ephrinA1 binding does not appear to simply engage the dimerization interface to cause EphA2 clustering, at the high EphA2 concentrations where the clustering interface is also engaged independently of ligand binding. Second, since the ability of the YSA peptide to antagonize ephrin binding suggests that the YSA peptide interacts with the ephrin-binding pocket of EphA2, it appears that m-ephrinA1 and YSA bind to the same binding pocket in the EphA2 ligand-binding domain, but induce the formation of distinctly different dimers stabilized through alternative interfaces. Because the YSA-binding site is on the opposite site of the clustering interface, the FRET results suggest that YSA likely exerts its dimer stabilizing effects via an allosteric mechanism as previously proposed^30, 41^. This mechanism may cause different changes compared to those induced by the binding of m-ephrinA1. Allosteric effects involving similar receptor regions but with opposite consequences can be also hypothesized for the R103E mutation, which inhibits not only ligand binding but also dimerization in the absence of ligand. Third, we observed EphA2 cluster formation in response to ephrinA1-Fc, demonstrating that the binding of this dimeric ligand to EphA2 is sufficient to induce EphA2 clustering. A long-standing view of some researchers in the field has been that soluble forms of the ephrin ligands have to be at least tetrameric in order to mimic the cell surface-anchored ephrins and activate Eph receptor signal transduction, whereas dimeric or monomeric ligands do not promote signaling and even function as antagonists^2, 24–27, 45^. Therefore, it is common to “pre-cluster” the dimeric ephrin-Fc proteins using anti-Fc antibodies in order to obtain multimeric forms of the ligands to induce Eph receptor clustering^2^. However, our FRET data show that unclustered, dimeric ephrinA1-Fc can induce EphA2 oligomers that are larger than dimers. This result is consistent with prior findings that EphA2, unlike some other Eph receptors, can be potently activated by dimeric ephrinA1-Fc^22^, which may depend on the high clustering propensity of EphA2 interfaces^18, 19, 46^.

Our quantitative FRET data explain, for the first time to our knowledge, how monomeric ephrins and engineered peptide ligands can act as EphA2 agonists. The mechanism involves promoting EphA2 dimerization, which is sufficient to induce EphA2 kinase activity. They also pose new intriguing questions, since currently it is not known if the signals initiated by different types of EphA2 oligomers are fundamentally different, with differently assembled oligomers engaging different downstream effectors. Alternatively, or in addition, the difference may be quantitative, with the strength of the signals scaling with the size and type of EphA2 oligomers. This can now be investigated, with the help of the mutants characterized in this work, and by direct comparisons of EphA2 signaling pathways activated in response to different ligands. There are already some indications that the response of EphA2 to different ligands may be different. For example, it has been reported that m-ephrinA1 activates EphA2 with slower kinetics than ephrinA1-Fc^12^, that Eph receptor oligomers of different sizes bind different cellular effectors and trigger different cellular responses, and that the tyrosine phosphorylation pattern of an Eph receptor and its downstream signaling network utilization depend on the nature of the ephrin ligand^47–51^. These findings support our hypothesis that different EphA2 assemblies can have distinctive signaling properties. It is further conceivable that different EphA2 assemblies are trafficked through different endosomal routes, and some evidence supports the notion that EphA2 signaling from different cellular compartments can lead to different functional consequences^52–55^. For example, phosphosite specific phosphotyrosine phosphatases differentially localized in the various endosomal compartments could differentially affect the pattern of EphA2 phosphorylated residues and, therefore, downstream signaling^55–59^. In conclusion, our biophysical characterization of EphA2 oligomerization in live cells suggests that different EphA2 ligands may be linked to selective receptor signaling functions by shaping the assembly of the EphA2 complexes through distinct interfaces.

## Methods

### Plasmid constructs

For all constructs, we used the pcDNA3.1 (+) vector (Invitrogen) for expression in mammalian cells. The EphA2 constructs consist of EphA2, a flexible 15 amino acid linker (GGS)_5_, and either mTurquoise or eYFP at the C terminus^37^. The EphA2 G131Y, R103E and L223R/L254R/V255R mutants were generated using the QuikChange II site-directed mutagenesis kit (Agilent Technologies, Santa Clara, CA), following the manufacturer’s recommended protocol. To generate the L223R/L254R/V255R EphA2 mutant, we consecutively engineered the L223R mutation, the L254R mutation, and the V255R mutation as described^37^. All primers used to generate the plasmids used in this study are shown in Supplementary Table 4.

### Cell culture and transfection

HEK293T cells were purchased from American Type Culture Collection (Manassas, VA, USA). 24 h before transfection, the cells were cultured on collagen-coated, glass bottom 35 mm Petri dishes (MatTek Corporation, MA) in Dulbecco’s modified Eagle medium supplemented with 10% fetal bovine serum (FBS, Hyclone), 3.5 g/L (19.4 mM) D-glucose and 1.5 g/L (17.9 mM) sodium bicarbonate at 37 °C in a 5% CO_2_ environment. The cells were co-transfected with mixtures of each EphA2 mutant tagged with mTurq or eYFP using Lipofectamine 3000 (Invitrogen) according to the manufacturer’s recommended protocol. Twelve hours after transfection, the cells were serum starved for at least 12 h. Before imaging, the medium was replaced with hypo-osmotic medium (1:9 serum-free medium: H_2_O; 25 mM HEPES) to induce swelling, as described previously^31^. The cells were allowed to settle for 10 min and imaged for approximately 2 h.

### Two photon microscopy of cells under reversible osmotic stress

Imaging was performed using a two photon microscope equipped with the OptiMis True Line Spectral Imaging system (Aurora Spectral Technologies, WI). The details of the microscope have been described previously^32, 60^. In brief, a Mai Tai laser (Spectra-Physics, Santa Clara) that generates femtosecond mode locked pulses at wavelengths between 690 and 1040 nm was used as excitation source. Two images for each cell were acquired: one at 800 nm to primarily excite the donor fluorophore, and another at 960 nm to primarily excite the acceptor. Experiments were performed in HEK293T cells under reversible osmotic stress. The reversible osmotic swelling was necessary because the cell membrane is normally highly “wrinkled”, and the reversible osmotic stress eliminates these wrinkles. Thus, the effective 3D protein concentration, determined using purified fluorescent protein standards of known concentration, can be converted into 2D receptor concentrations in the plasma membrane^20^. Only areas of plasma membrane not in contact with other cells were imaged to ensure that EphA2 receptors did not interact with ephrins from neighboring cells.

### Thermodynamic analysis of receptor association

$E_{\rm oligo},$ the FRET occurring due to the specific association of donor- and acceptor-labeled receptors, is modeled based on Raicu’s kinetic theory formalism^32^:

1 $$E_{\rm {oligo}} = \frac{{\mu _{\rm {oligo}}}}{{\left[ D \right]}}\mathop {\sum }\limits_{k = 1}^{n - 1} \frac{{k\left( {n - k} \right)\widetilde E}}{{1 + \left( {n - k - 1} \right)\widetilde E}}\left( {\begin{array}{*{20}{c}} n \\ k \end{array}} \right)x_D^kx_A^{n - k}$$

Here *n* represents the oligomer order, $\mu _{\rm {oligo}}$ is the concentration of oligomers, and [*D*] is the concentration of donors. $x_D$ and $x_A$ are the fraction of donors and acceptors, respectively, $x_D + x_A = 1$. ${\widetilde E}$ is the “Intrinsic FRET” or “pair-wise FRET efficiency” which primarily depends on the average distance between the fluorescent proteins in the oligomer, *d*, according to^21, 61^:

2 $$\widetilde E = \frac{1}{{1 + \left( {\frac{d}{{R_0}}} \right)^6}}$$

In Equation (2), *R*_0_ is the Förster radius of the FRET pair (in this case, 54.5 Å).

The FRET efficiency due to specific interactions in the oligomer is written as:

3 $$E_{\rm {oligo}} = \frac{{f_{\rm {oligo}}}}{{n \cdot x_D}} \cdot E$$

where $$E = \mathop {\sum }\limits_{k = 1}^{n - 1} \frac{{k\left( {n - k} \right)\widetilde E}}{{1 + \left( {n - k - 1} \right)\widetilde E}}\left( {\begin{array}{*{20}{c}} n \\ k \end{array}} \right)x_D^kx_A^{n - k}\cdot{f_{\rm {oligo}}}$$ is the fraction of proteins in the oligomeric state, and depends of the association constant *K* and the total receptor concentration, [*T*] = [*D*] + [*A*], according to equation (4)

4 $$f_{\rm oligo} = \frac{{n\mu _{\rm {oligo}}}}{{[T]}} = \frac{{nK[m]^n}}{{[T]}}$$

Since there is no analytic form for the proximity FRET when the size of the fluorophores is non-negligible, it is simulated for all $n = 2{:}6$ over a gridded multidimensional space of the two adjustable parameters: $\widetilde E$, and *K*, for acceptor concentrations ranging from zero to $8 \times 10^{ + 3}$ acceptors per μm^2^. The total FRET efficiency is calculated using Equations (3) and (4), while accounting for the so-called proximity FRET as described^21, 62^. The calculated FRET efficiency for different values of $\widetilde E$ and *K* is compared to the experimental one, and the MSE is calculated. This procedure is performed for each *n*. The value of *n* for which the MSE is minimized gives the best-fit oligomer model. Then, the best-fit proximity FRET model for the best-fit *n* is fixed as determined in the gridded search, and the values of $\widetilde E$ and *K*, and their 95% confidence intervals are determined using non-linear least square fitting. This fitting procedure was recently tested and verified, as described in detail in ref. ^21^.

The stability of the dimer is related to the dissociation constant *K*_diss_ = 1/*K* according to:

5 $$\Delta G = RT\ln K_{\rm {diss}}$$

with the standard state defined as $K_{\rm {diss}}^0 = 1$ receptor per nm^2^.

### Cell migration assays

To assess the migratory ability of the cells, the CytoSelect™ Cell Haptotaxis Assay Kit (CellBiolabs, CA) was used according to the manufacturer’s recommended protocol with some modifications as follows. HEK 293T cells were seeded at a density of 3.5 × 10^5^ cells per well in 6-well plates. The cells were transfected with the plasmids, cultured for 24 h and serum starved for 12 h. The cells were suspended at a concentration of 1 × 10^6^ cells per ml in 0.5% BSA in serum-free medium (in some cases supplemented with m-ephrinA1) and 0.2 ml were added to each insert of the Transwell provided with the kit, which contains a polycarbonate membrane with pore size of 8 μm, coated with collagen I on the lower side. Inserts were incubated for 4 h at 37 °C in the plate containing 0.5 ml medium with 10% FBS. Serum-free medium was aspirated from the inserts and the upper side of the polycarbonate membrane inside the inserts was cleaned with cotton swabs in order to remove the cells that had not migrated across the membrane. The inserts were placed in new clean wells with 0.3 ml 1 × Lysis Buffer/CyQuant® GR dye and incubated for 10 min at room temperature. To measure the fluorescence of the dye solution at 480 nm/520 nm, 0.2 ml of the lysate were placed into the well of a new 96-well plate. The output of this assay is fluorescence intensity, which is directly proportional to the number of cells that have migrated through the polycarbonate membrane.

### Western blots

Cells were transfected with plasmids using Lipofectamine 3000, grown for about 12 h, serum-starved for ~12 h and treated with FBS for 15 min, in some cases in the presence of 5 μg/ml (200 nM) m-ephrinA1. Cell lysates were collected with lysis buffer (25 mM Tris-Cl, 0.5% TritonX-100, 20 mM NaCl, 2 mM EDTA and phosphatase and protease inhibitors (Roche Applied Science)). The lysates were centrifuged at 14,000 × *g* for 15 min at 4 °C and stored at −20 °C. BCA assays (Bio-Rad) were used to measure the protein concentrations of the samples. Lysates mixed with LDS sample buffer and reducing buffer were run on 3–8% NuPAGE^H^Novex^H^Tris-Acetate mini gels (Invitrogen, CA) and transferred onto nitrocellulose membranes. The membranes were blocked with 5% non-fat milk in 1 × TBST. EphA2 expression was quantified using anti-EphA2 antibodies (Cell Signalling, MA). S897 and Y772 phosphorylation levels were quantified using anti-phospho-Ser897 and anti-phospho-Tyr772 antibodies (Cell Signaling, MA) followed by an anti-rabbit HRP conjugated antibody (Promega, WI). Nitrocellulose membranes were incubated for 2 min with Amersham ECL Plus Western Blotting Detection Reagent (GE Health Care Life Sciences, PA) and exposed from 1 to 60 s to capture images with the ChemiDoc imaging system (Bio-Rad, CA). Uncropped images of all Western blots presented in the study are shown in Supplementary Figures 13-18.

### Code availability

A commercial code supporting OptiMiS spectral acquisition is available from Aurora. The custom code for the OptiMiS spectral acquisition analysis used in this work is available from Dr. Christopher King on reasonable request. There are no restrictions on code availability.

### Data availability

All data generated or analyzed during this study are included in this published article (and its Supplementary Information files), and https://figshare.com/projects/The_EphA2_receptor_is_activated_through_induction_of_distinct_ligand-dependent_oligomeric_structures/28470. There are no restrictions on data availability.

## Electronic supplementary material

Supplementary Information
