# Author Correction: The EphA2 receptor is activated through induction of distinct, ligand-dependent oligomeric structures

**DOI:** 10.1038/s42003-018-0044-4

**Published:** 2018-04-19

**Authors:** Deo R. Singh, Pranjali Kanvinde, Christopher King, Elena B. Pasquale, Kalina Hristova

**Affiliations:** 1 Department of Materials Science and Engineering, Johns Hopkins University, 3400 Charles Street, Baltimore, MD 21218 USA; 2 Institute of NanoBioTechnology, Johns Hopkins University, 3400 Charles Street, Baltimore, MD 21218 USA; 3 Program in Molecular Biophysics, Johns Hopkins University, 3400 Charles Street, Baltimore, MD 21218 USA; 4 Sanford Burnham Prebys Medical Discovery Institute, 10901 North Torrey Road, La Jolla, CA 92037 USA; 5 Pathology Department, University of California San Diego, La Jolla, CA 92093 USA

Correction to: *Communications Biology* 10.1038/s42003-018-0017-7, published online 22 February 2018.

The original published version of the paper contained labelling errors in Figures 1 and 5. The *x*-axis of Figure 1e was incorrectly labelled as “Receptors . μm^2^” instead of “Receptors . μm^−2^”. The figure key in Figure 5b incorrectly labelled the blue circles as “Wild-type” and the purple circles as “+m-ephrinA1”. The correct labels are blue: “Wild-type+m-ephrinA1” and purple: “Wild-type, no ligand.” The original version also contained a typesetting error in Table 1. The values in the second row, rightmost column, were not all set in bold text as required. The errors do not impact the conclusions in the main text of the paper. These errors have been corrected in the PDF and HTML versions of the paper.

