# Supplementary material for: The EphA2 receptor is activated through induction of distinct, ligand-dependent oligomeric structures
Source: Commun Biol. 2018 Feb 22;1:15. doi: 10.1038/s42003-018-0017-7 (PMC6123813; doi:10.1038/s42003-018-0017-7)
Supplement: Supplementary file 1 — Supplementary Information [file 42003_2018_17_MOESM1_ESM.pdf]

Supplementary Table 1. Parameters describing unliganded EphA2 dimerization

EphA2 construct	K_{diss} (receptors.µm^{-2})	ΔG (kcal.mole $^{-1}$)	$\tilde{E}$	d (Å)
wild-type	206 (133 to 278)	-5.0 (-4.8 to -5.3)	0.44 (0.41 to 0.47)	57 (56 to 58)
L223R/L254R/L255R	1100 (628 to 1582)	-4.0 (-3.8 to -4.4)	0.49 (0.42 to 0.56)	55 (52 to 58)
G131Y	230 (122 to 338)	-5.0 (-4.7 to -5.3)	0.31 (0.28 to 0.33)	62 (61 to 64)
R103E	1200 (851 to 1564)	-4.0 (-3.8 to -4.2)	0.46 (0.42 to 0.51)	56 (54 to 58)

K_{diss} is the dissociation constant (receptors per µm^2); ΔG is the dimerization free energy; $\tilde{E}$ is the intrinsic FRET efficiency; and d is the calculated distance between the fluorescent proteins in the EphA2 dimers. K_{diss} and $\tilde{E}$ are determined from the fit of the dimerization model to the FRET data, and the uncertainties are the 95% confidence intervals from the fit. ΔG and d are calculated using Equations 5 and 2, respectively.

Supplementary Table 2. Parameters describing EphA2 dimerization in the presence of m-ephrinA1

EphA2 construct	K_{diss} (receptors.μm^{-2})	ΔG (kcal.mole^{-1})	$\tilde{E}$	d (Å)
wild-type	11 (5 to 18)	-6.7 (-6.5 to -7.2)	0.45 (0.44 to 0.47)	56 (55 to 57)
L223R/L254R/L255R	14 (2 to 25)	-6.6 (-6.3 to -7.8)	0.36 (0.35 to 0.37)	60 (59 to 61)
G131Y	48 (32 to 64)	-5.9 (-5.7 to -6.1)	0.45 (0.44 to 0.47)	56 (55 to 57)
R103E	500 (320 to 680)	-4.5 (-4.3 to -4.8)	0.56 (0.51 to 0.61)	52 (51 to 54)

K_{diss} is the dissociation constant (receptors per μm^2); ΔG is the dimerization free energy; $\tilde{E}$ is the intrinsic FRET efficiency; and d is the calculated distance between the fluorescent proteins in the EphA2 dimers. K_{diss} and $\tilde{E}$ are determined from the fit of the dimerization model to the FRET data, and the uncertainties are the 95% confidence intervals determined in the fit. ΔG and d are calculated using Equations 5 and 2, respectively.

Supplementary Table 3. Parameters describing EphA2 dimerization in the presence of the YSA peptide

EphA2 construct	K_{diss} (receptors.µm^{-2})	ΔG (kcal.mole^{-1})	$\tilde{E}$	d (Å)
wild-type	69 (46 to 92)	-5.7 (-5.5 to -5.9)	0.52 (0.5 to 0.54)	54 (53 to 55)
L223R/L254R/L255R	428 (325 to 530)	-4.6 (-4.5 to -4.8)	0.54 (0.51 to 0.57)	53 (52 to 54)
G131Y	71 (41 to 101)	-5.7 (-5.5 to -6.0)	0.49 (0.46 to 0.51)	55 (54 to 56)
R103E EphA2	319 (206 to 432)	-4.8 (-4.6 to -5.0)	0.42 (0.39 to 0.45)	58 (56 to 59)

K_{diss} is the dissociation constant (receptors perµm^2); ΔG is the dimerization free energy; $\tilde{E}$ is the intrinsic FRET efficiency; and d is the calculated distance between the fluorescent proteins in the EphA2 dimers. K_{diss} and $\tilde{E}$ are determined from the fit of the dimerization model to the FRET data, and the uncertainties are the 95% confidence intervals from the fit. ΔG and d are calculated using Equations 5 and 2, respectively.

Supplementary Table 4: Primers used to generate the EphA2 mutants used in the study

Primer name	5'-3' sequence
G131Y_ F	GAGTCGGACCTGGACTACTACACCAACTTCCAGAAGCGC
G131Y_ R	GCGCTTCTGGAAGTTGGTGTAGTAGTCCAGGTCCGACTC
R103E _F	GCTCAAGTTTACTGTAGAAGACTGCAACAGCTTCC
R103E_ R	GGAAGCTGTTGCAGTCTTCTACAGTAAACTTGAGC
L223R_ F[a]	GGCTCTGATGCACCTTCCCGGGCCACTGTGGCCGG
L223R_ R[a]	CCGGCCACAGTGGCCCGGGAAGGTGCATCAGAGCC
L254R _F[a]	GCAGTGGATGGCGAGTGGCGGGTGCCCATTGGGCAGTGCC
L254R_ R[a]	GGCACTGCCCAATGGGCACCCGCCACTCGCCATCCACTGC
V255R_ F[a]	GCAGTGGATGGCGAGTGGCGGCGGCCCATTGGGCAGTGCC
V255R_ R[a]	GGCACTGCCCAATGGGCCGCCGCCACTCGCCATCCACTGC

[a]The cloning of the L223R/L254R EphA2 mutant was described in[1]

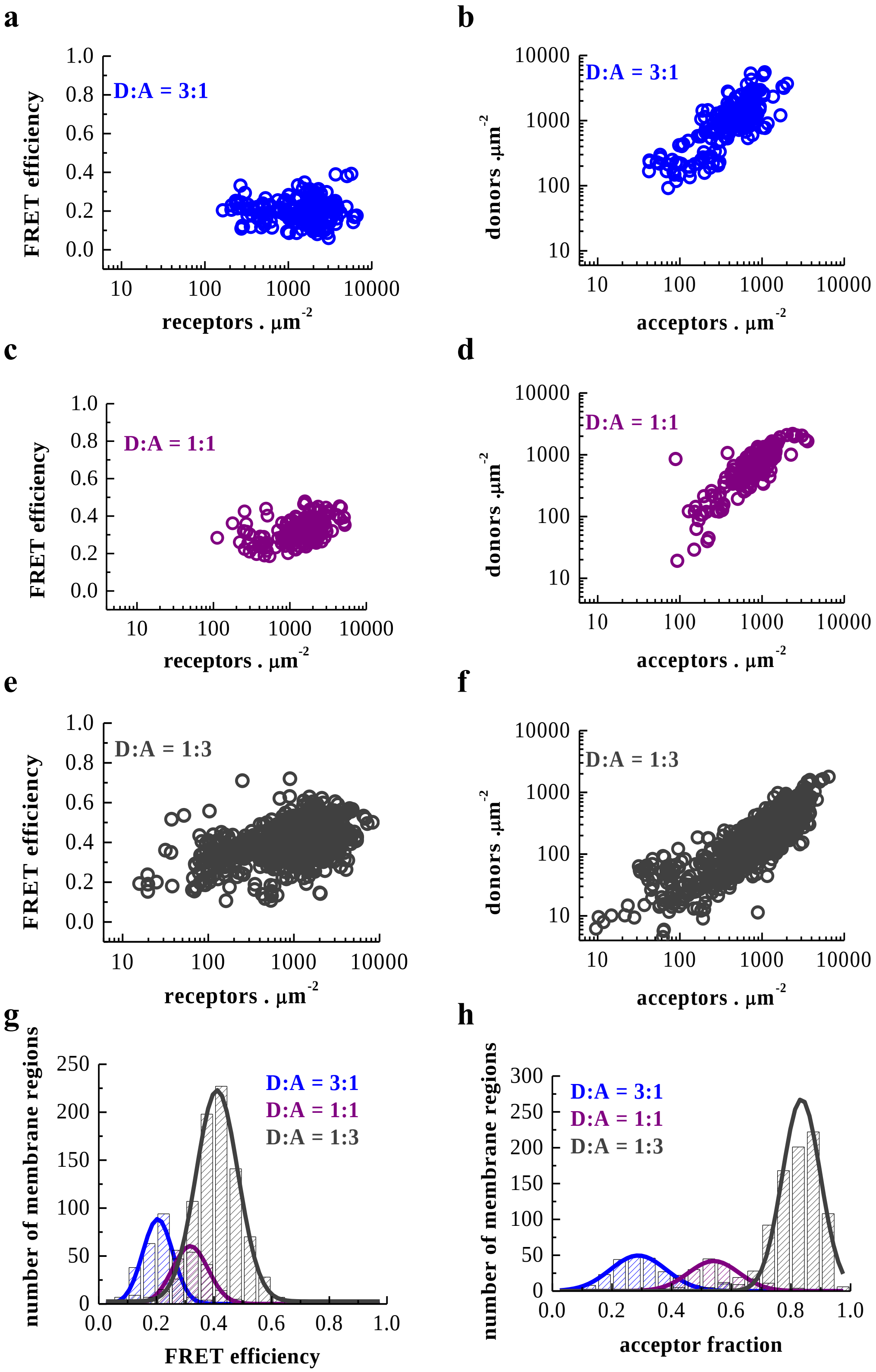
a
b
c
d
e
f
g
h
D:A = 3:1
D:A = 1:1
D:A = 1:3
FRET efficiency
receptors . μm-2
donors .μm-2
acceptors . μm-2
number of membrane regions
acceptor fraction

Supplementary Figure 1. The EphA2 receptor forms clusters in response to ephrinA1-Fc. (a), (c), (e) FRET efficiencies *versus* total receptor concentration for HEK293T cells co-transfected with DNA encoding EphA2-mTurq (donor) and EphA2-YFP (acceptor) in 3:1, 1:1, and 1:3 ratios. The cells were stimulated with 50 nM ephrinA1-Fc. (**b), (d), (f)** donor concentration *versus* acceptor concentration for the same cells. In total, 50 cells were imaged to obtain 239 data points in (a) and (b), 50 cells to obtain 186 data points in (c) and (d), and 275 cells to obtain 858 data points in (e) and (f). (**g)** Histograms of the FRET efficiencies in (a), (c), and (e). The histograms were fitted with Gaussian functions to obtain the mean FRET efficiencies and the standard errors. Only data points above 100 receptors per μm^2 were included, because in this case the FRET efficiency depends primarily on the donor-to-acceptor ratio but not on the total concentration. (**h)** Histograms of the acceptor fractions in (a), (c), and (e) for total EphA2 concentrations exceeding 100 receptors per μm^2. The histograms were fitted with Gaussian functions to obtain the mean acceptor fractions and the standard errors. The FRET efficiencies are plotted against the acceptor fractions in **Figure 1f.**

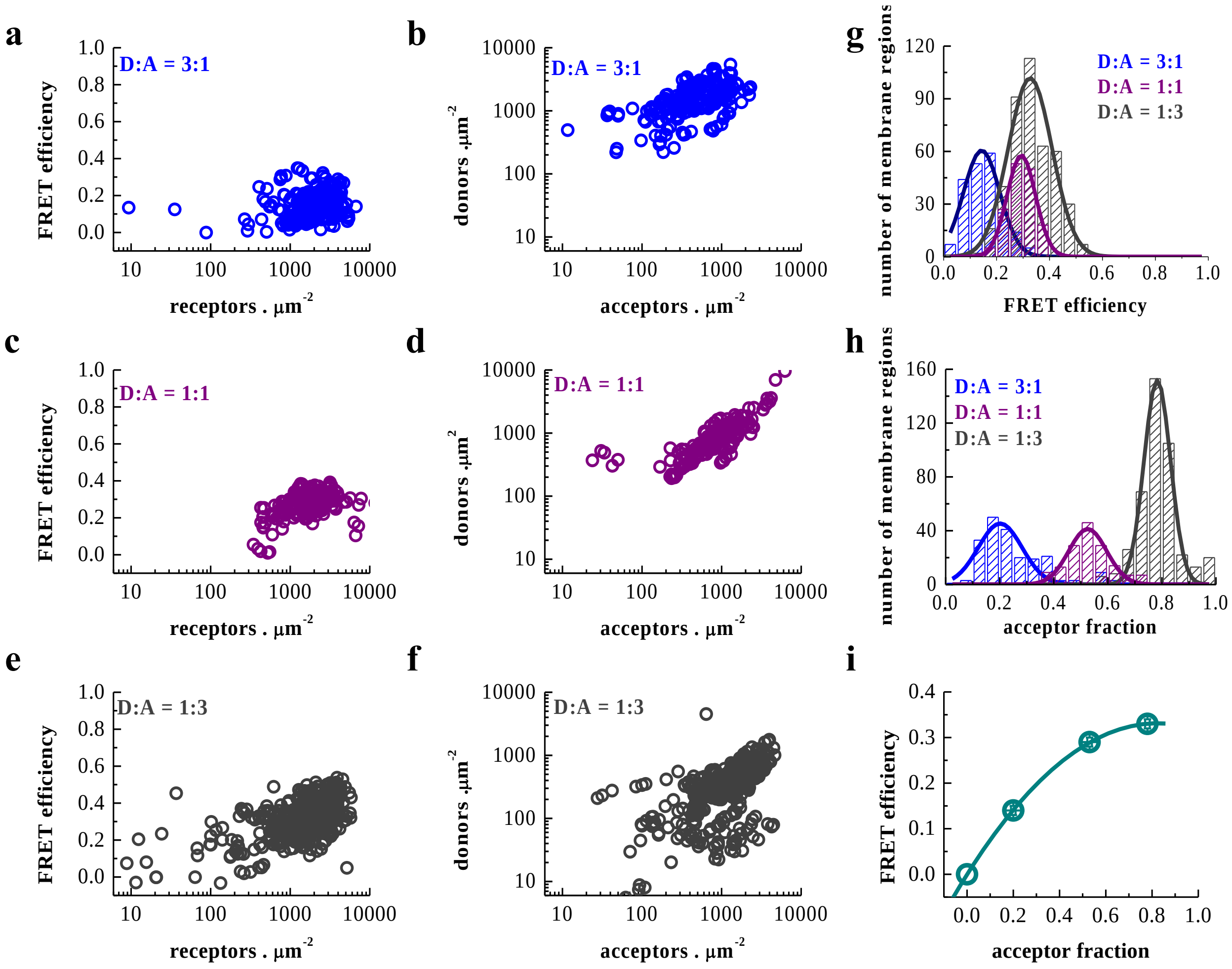

Supplementary Figure 2. The EphA2 L223R/L254R/V255R mutant forms clusters in response to ephrinA1-Fc. (a), (c), (e) FRET efficiencies *versus* total receptor concentration for HEK293T cells co-transfected with DNA encoding EphA2-mTurq L223R/L254R/V255R (donor) and EphA2-eYFP L223R/L254R/V255R (acceptor) in 3:1, 1:1, and 1:3 ratios. The cells were stimulated with 50 nM ephrinA1-Fc. (**b), (d), (f)** donor concentration *versus* acceptor concentration for the same cells. 50 cells were imaged to obtain 189 data points in (c) and (d), and 196 cells were imaged to obtain 563 data points in (e) and (f). (**g)** Histograms showing the FRET efficiencies in (a), (c), and (e) acquired when the total EphA2 concentration exceeded 1,000 receptors per μm^2. The histograms were fitted with Gaussian functions to obtain the mean FRET efficiencies and the standard errors. (**h)** Histograms showing the acceptor fractions in (a), (c), and (e) for total EphA2 concentrations exceeding 1,000 receptors per μm^2, were fitted with Gaussian functions to obtain the mean acceptor fractions and the standard errors. **(i)** The FRET efficiencies are plotted against the acceptor fractions along with the standard errors (smaller than the symbol size). The dependence is non-linear, indicative of clustering[2].

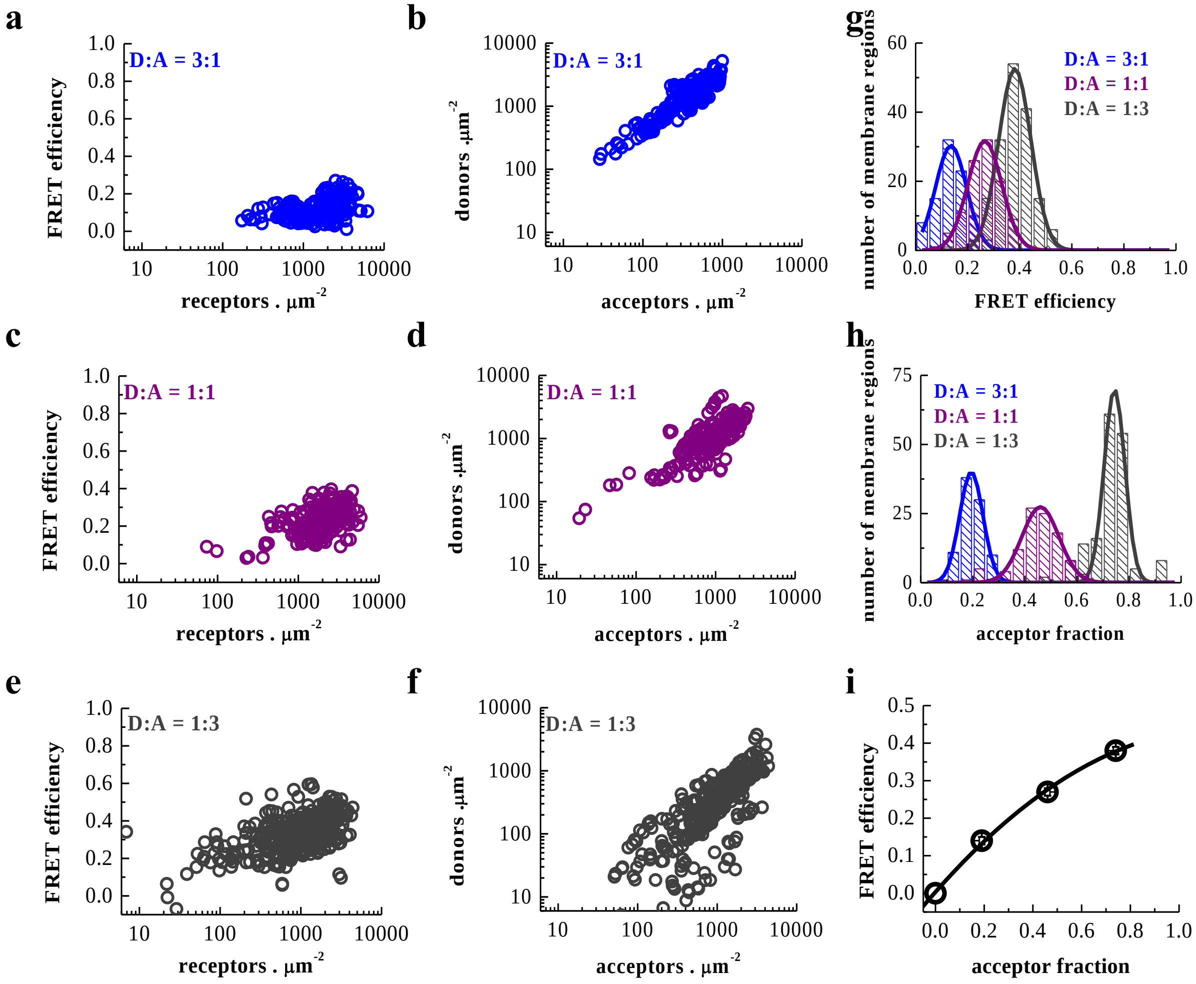

Supplementary Figure 3. The EphA2 R103E mutant forms clusters in response to ephrinA1-Fc. (a), (c), (e) FRET efficiencies *versus* total receptor concentration for HEK293T cells co-transfected with DNA encoding EphA2-mTurq R103E (donor) and EphA2-eYFP R103E (acceptor) in 3:1, 1:1, and 1:3 ratios. The cells were stimulated with 50 nM ephrinA1-Fc. **(b), (d), (f)** donor concentration *versus* acceptor concentration for the same cells. 50 cells were imaged to obtain 221 data points in (a) and (b), 50 cells were imaged to obtain 217 data points in (c) and (d), and 201 cells were imaged to obtain 474 data points in (e) and (f). **(g)** Histograms of the FRET efficiencies in (a), (c), and (e) acquired when the total EphA2 concentration exceeded 1,000 receptors per μm^2. The histograms were fitted with Gaussian functions to obtain the mean FRET efficiencies and the standard errors. **(h)** Histograms of the acceptor fractions in (a), (c), and (e) for total EphA2 concentrations exceeding 1,000 receptors per μm^2, were fitted with Gaussian functions to obtain the mean acceptor fractions and the standard errors. **(i)** The FRET efficiencies are plotted against the acceptor fractions along with the standard errors (smaller than the symbol size). The dependence is non-linear, indicative of clustering[2].

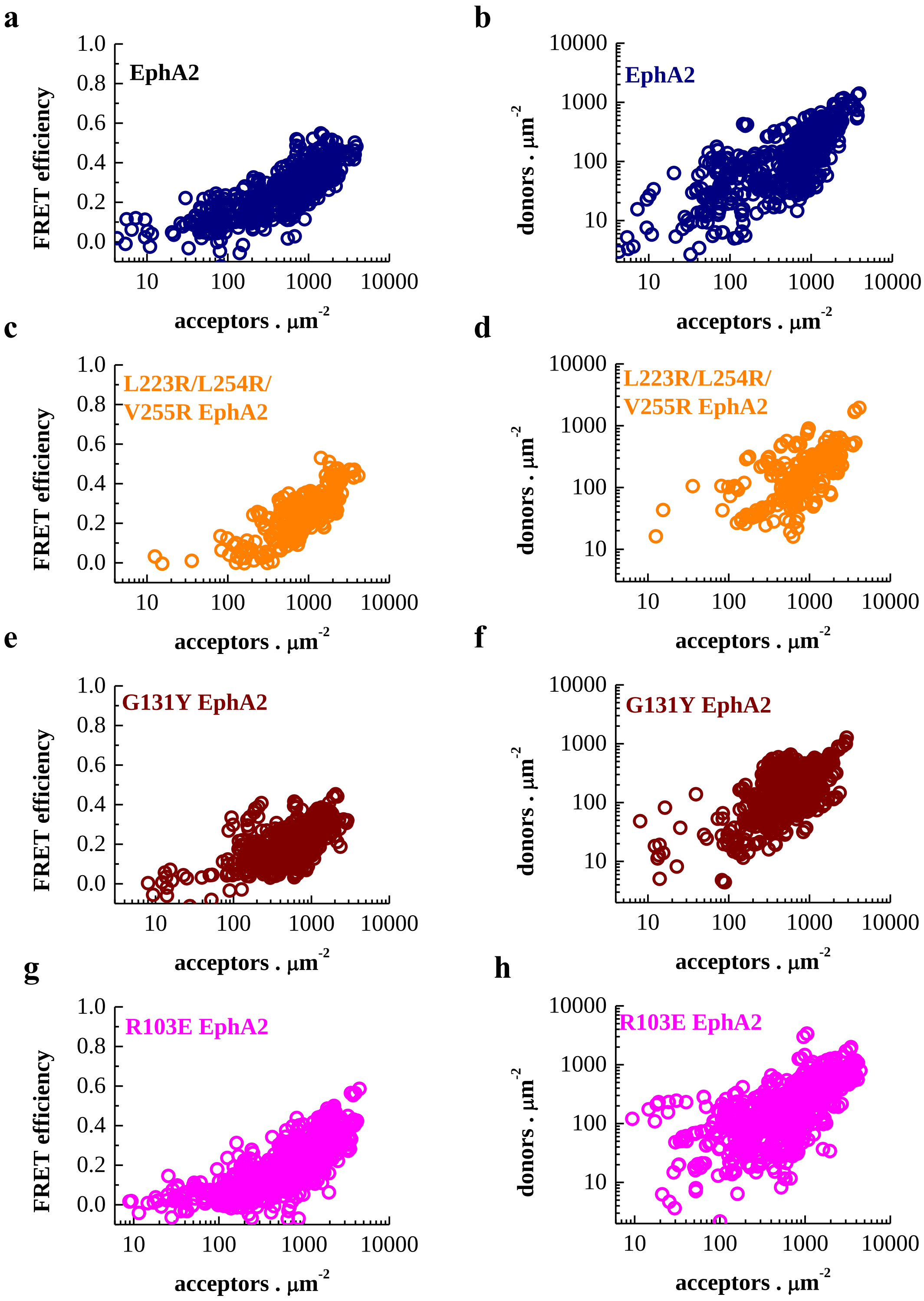
a
EphA2
FRET efficiency
acceptors . μm^{-2}
b
EphA2
donors . μm^{-2}
acceptors . μm^{-2}
c
L223R/L254R/
V255R EphA2
FRET efficiency
acceptors . μm^{-2}
d
L223R/L254R/
V255R EphA2
donors . μm^{-2}
acceptors . μm^{-2}
e
G131Y EphA2
FRET efficiency
acceptors . μm^{-2}
f
G131Y EphA2
donors . μm^{-2}
acceptors . μm^{-2}
g
R103E EphA2
FRET efficiency
acceptors . μm^{-2}
h
R103E EphA2
donors . μm^{-2}
acceptors . μm^{-2}

Supplementary Figure 4. FRET data for EphA2 wild-type and the L223R/L254R/V255R, G131Y and R103E mutants in the absence of ligand. HEK293T cells were co-transfected with DNA encoding donors and acceptors in a 1:3 ratio. **(a), (c), (e), (g)** FRET *versus* acceptor concentration. **(b), (d), (f), (h)** donor concentration versus acceptor concentration. 151 cells were imaged to obtain 576 data points in (a) and (b), 200 cells were imaged to obtain 289 data points in (c) and (d), 212 cells were imaged to obtain 905 data points in (e) and (f), and 300 cells were imaged to obtain 824 data points in (g) and (h).

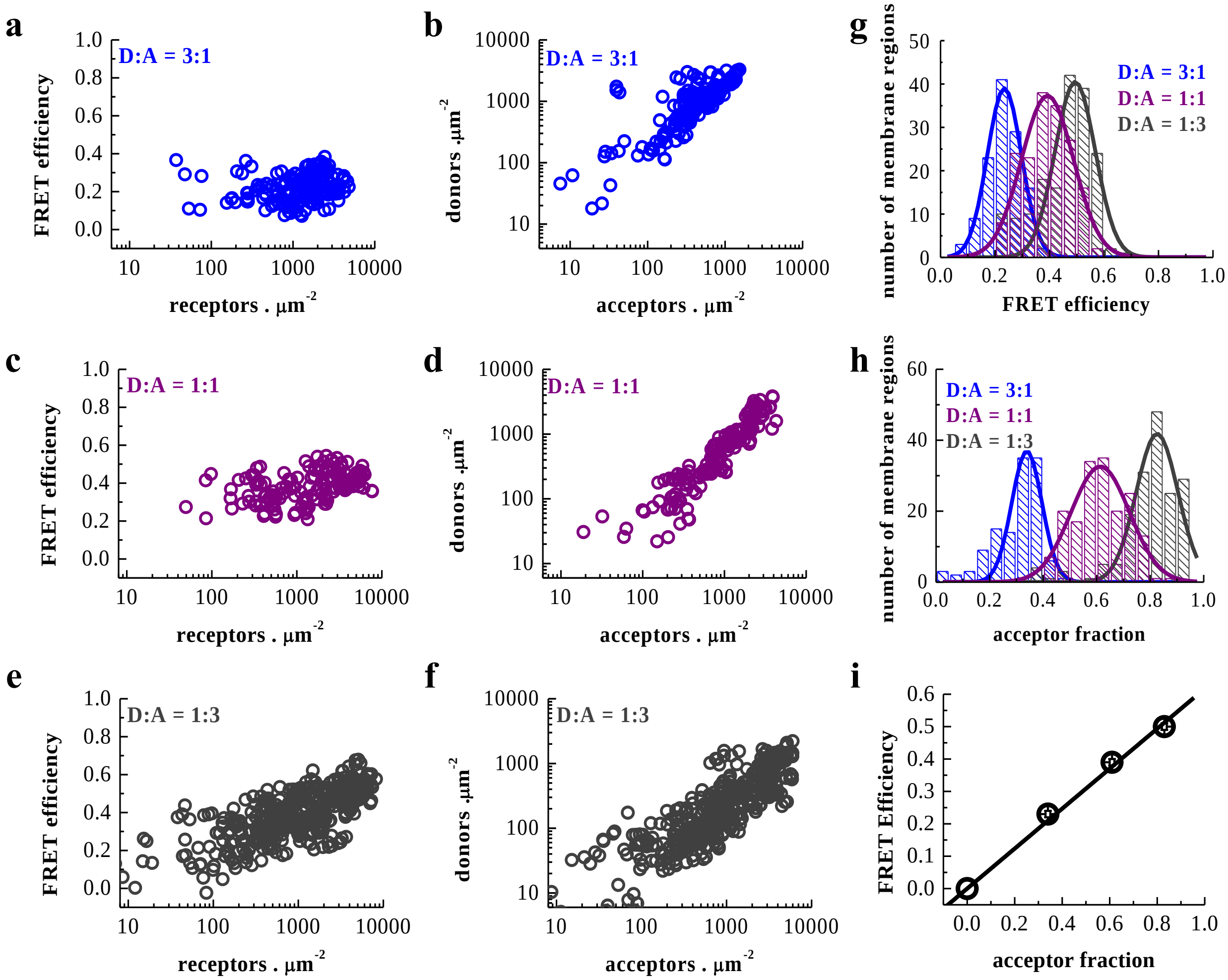

Supplementary Figure 5. Wild-type EphA2 forms dimers in response to m-ephrinA1. (a), (c), (e) FRET efficiencies *versus* total receptor concentration for HEK293T cells co-transfected with DNA encoding EphA2-mTurq (donor) and EphA2-eYFP (acceptor) in 3:1, 1:1, and 1:3 ratios. The cells were stimulated with 200 nM m-ephrinA1. **(b), (d), (f)** donor concentration *versus* acceptor concentration for the same cells. 50 cells were imaged to obtain 183 data points in (a) and (b), 50 cells were imaged to obtain 150 data points in (c) and (d), and 232 cells were imaged to obtain 440 data points in (e) and (f). **(g)** Histograms of the FRET efficiencies in **(a), (c),** and **(e)** acquired when the total EphA2 concentration exceeded 1,000 receptors per μm^2. The histograms were fitted with Gaussian functions to obtain the mean FRET efficiencies and the standard errors. (**h)** Histograms of the acceptor fractions in **(a), (c),** and **(e)** for total EphA2 concentrations exceeding 1,000 receptors per μm^2, were fitted with Gaussian functions to obtain the mean acceptor fractions and the standard errors. **(i)** The FRET efficiencies are plotted against the acceptor fractions along with the standard errors (smaller than the symbol size). The dependence is linear, indicative of dimer formation[2].

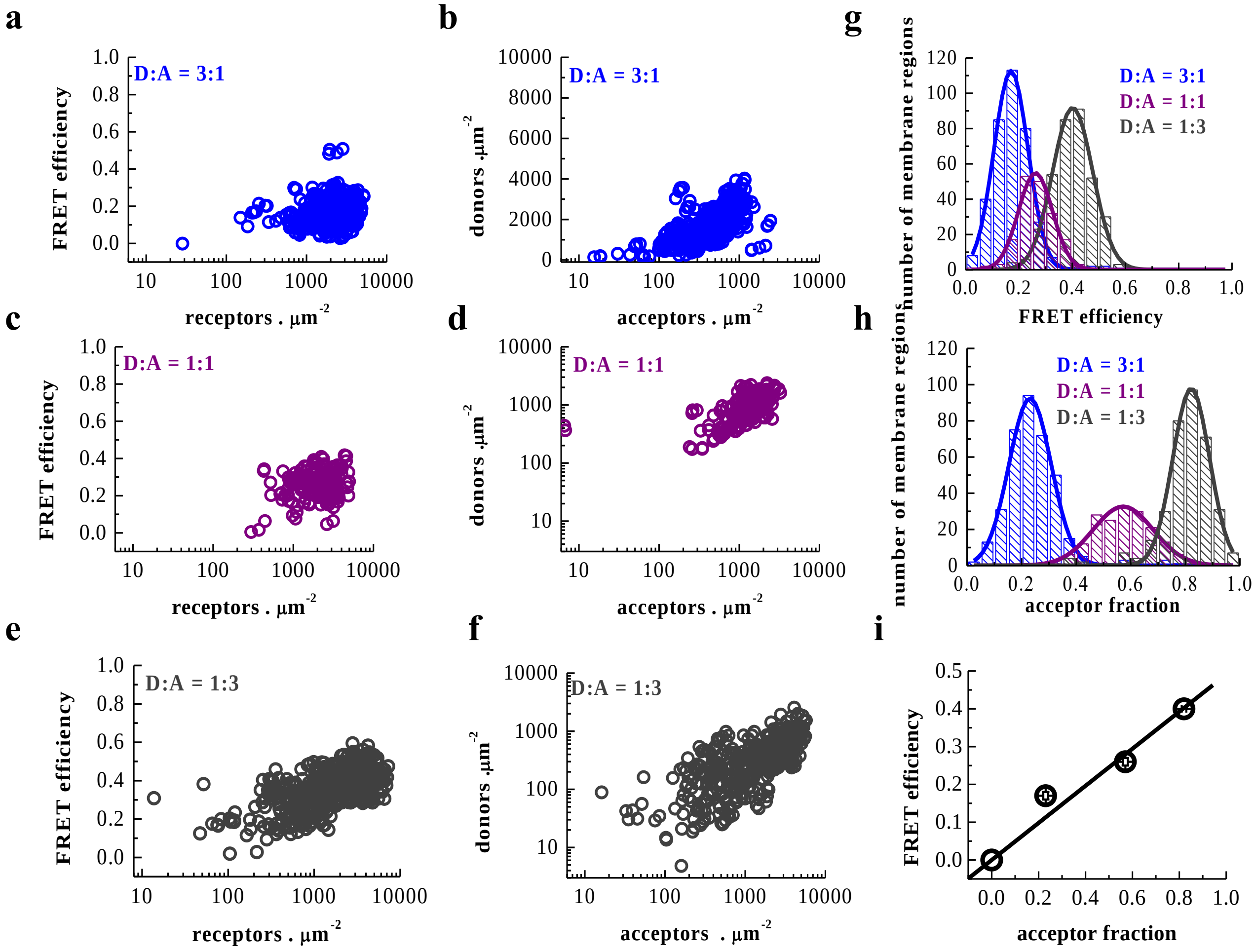

Supplementary Figure 6. The EphA2 L223R/L254R/V255R mutant forms dimers in response to m-ephrinA1. (a), (c), (e) FRET efficiencies *versus* total receptor concentration for HEK293T cells co-transfected with DNA encoding EphA2-mTurq L223R/L254R/V255R (donor) and EphA2-eYFP L223R/L254R/V255R (acceptor) in 3:1, 1:1, and 1:3 ratios. The cells were stimulated with 200 nM m-ephrinA1. **(b), (d), (f)** donor concentration *versus* acceptor concentration for the same cells. 100 cells were imaged to obtain 414 data points in (a) and (b), 50 cells were imaged to obtain 200 data points in (c) and (d), and 176 cells were imaged to obtain 531 data points in (e) and (f). **(g)** Histograms of the FRET efficiencies in **(a), (c),** and **(e)** acquired when the total EphA2 concentration exceeded 1,000 receptors per μm^2. The histograms were fitted with Gaussian functions to obtain the mean FRET efficiencies and the standard errors. **(h)** Histograms of the acceptor fractions in **(a), (c),** and **(e)** for total EphA2 concentrations exceeding 1,000 receptors per μm^2, were fitted with Gaussian functions to obtain the mean acceptor fractions and the standard errors. **(i)** The FRET efficiencies are plotted against the acceptor fractions along with the standard errors (smaller than the symbol size). The dependence is linear, indicative of dimer formation[2].

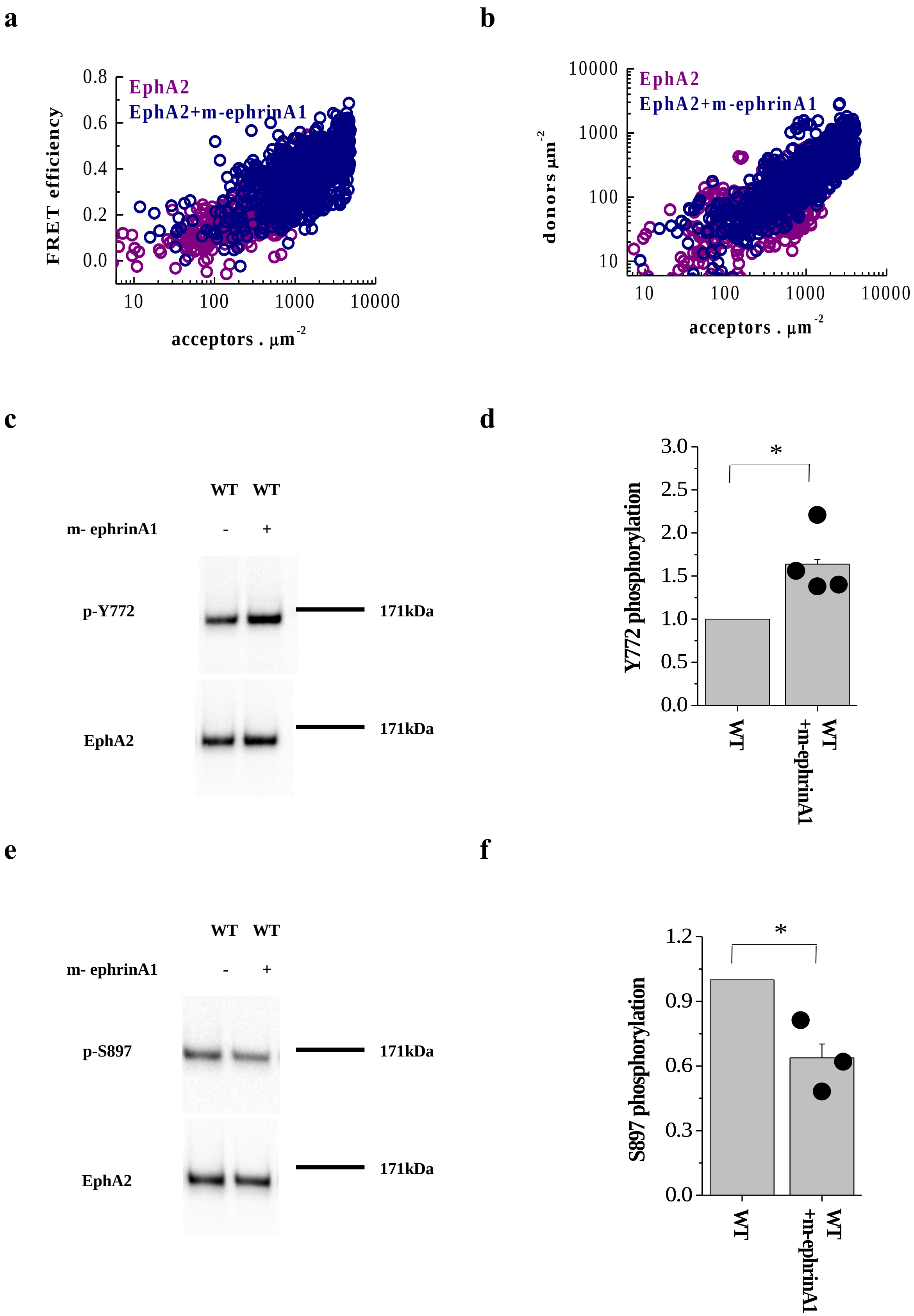
a
FRET efficiency
EphA2
EphA2+m-ephrinA1
0.8
0.6
0.4
0.2
0.0
10
100
1000
10000
acceptors . μm-2
b
donors μm-2
EphA2
EphA2+m-ephrinA1
10000
1000
100
10
acceptors . μm-2
c
WT WT
m- ephrinA1 - +
p-Y772
171kDa
EphA2
171kDa
d
Y772 phosphorylation
*
3.0
2.5
2.0
1.5
1.0
0.5
0.0
WT
WT +m-ephrinA1
e
WT WT
m- ephrinA1 - +
p-S897
171kDa
EphA2
171kDa
f
S897 phosphorylation
*
1.2
0.9
0.6
0.3
0.0
WT
WT +m-ephrinA1

Supplementary Figure 7. **Response of EphA2 wild-type to m-ephrinA1. (a)** FRET efficiency *versus* acceptor concentration with and without 200 nM m-ephrinA1 stimulation. **(b)** Donor concentration *versus* acceptor concentration with and without m-ephrinA1 stimulation. 151 cells were imaged to obtain 575 data points in the absence of m-ephrinA1 and 233 cells were imaged to obtain 440 data points in the presence of m-ephrinA1. (**c**) Representative western blots showing EphA2 Y772 phosphorylation with and without m-ephrinA1 stimulation. (**d**) Quantification of Y772 phosphorylation from four independent experiments (solid circles). Shown are means and standard errors**.** M-ephrinA1 enhances tyrosine phosphorylation. (* for $p < 0.05$ from Student's t-test). (**e**) Representative western blots showing EphA2 S897 phosphorylation with and without m-ephrinA1 stimulation. (**f**) Quantification from three independent experiments (solid circles), yielding means and standard errors. M-ephrinA1 reduces S897 phosphorylation. (* for $p < 0.05$ from Student's t-test).

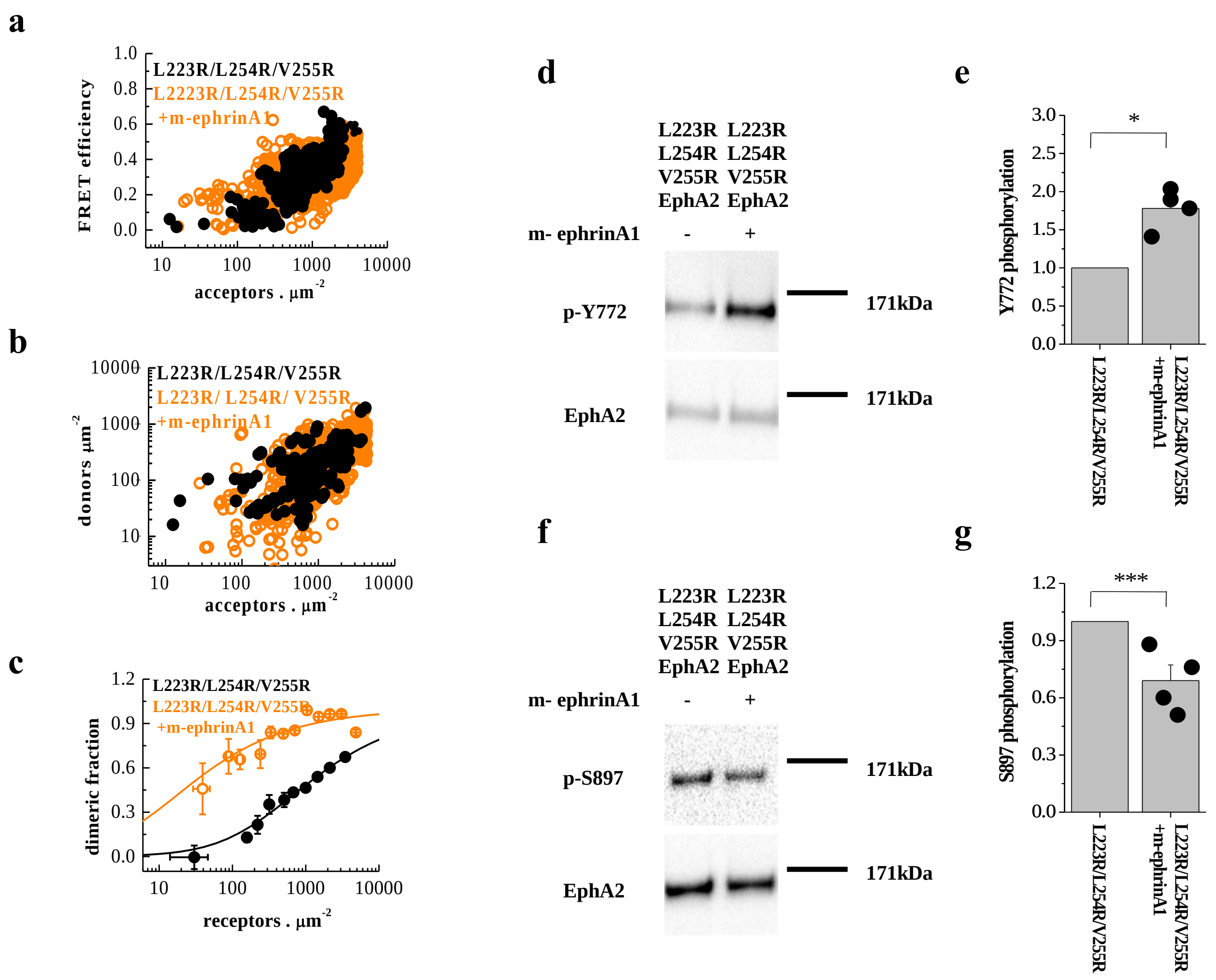

Supplementary Figure 8. **Response of the EphA2 L223R/L254R/V255R mutant to m-ephrinA1. (a)** FRET efficiency *versus* acceptor concentration with and without 200 nM m-ephrinA1 stimulation. **(b)** Donor concentration *versus* acceptor concentration with and without m-ephrinA1. 200 cells were imaged to obtain 289 data points in the absence of m-ephrinA1 and 176 cells were imaged to obtain 530 data points in the presence of m-ephrinA1 in (a) and (b). **(c)** Dimerization curves for EphA2 L223R/L254R/V255R with and without m-ephrinA1 stimulation; m-ephrinA1 enhances the dimerization of EphA2 L223R/L254R/V255R. **(d)** Representative western blots showing EphA2 L223R/L254R/V254R Y772 phosphorylation with and without m-ephrinA1 stimulation. (**e**) Quantification of Y772 phosphorylation from four independent experiments (solid circles). Shown are means and standard errors**.** M-ephrinA1 enhances tyrosine phosphorylation. (*for $p < 0.05$ from Student's t-test). (**f**) Representative western blots showing EphA2 L223R/L254R/V254R S897 phosphorylation with and without m-ephrinA1 stimulation. (**g**) Quantification from four independent experiments (solid circles), showing the means and standard errors. M-ephrinA1 reduces S897 phosphorylation. (*** for $p < 0.001$ from Student's t-test).

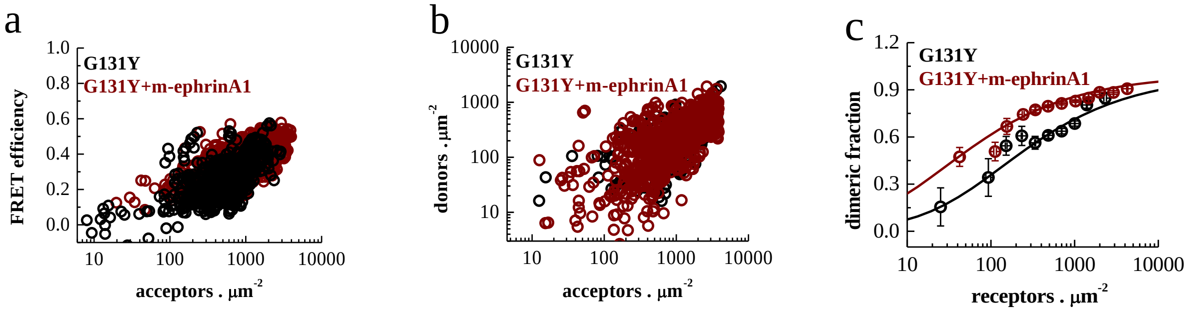

Supplementary Figure 9. FRET data for the EphA2 G131Y mutant, in the absence of ligand and in the presence of 200 nM m-ephrinA1. HEK293T cells were co-transfected with DNA encoding donors and acceptors in a 1:3 ratio. (**a**) FRET efficiency *versus* acceptor concentration. (**b**) Donor concentration *versus* acceptor concentration. (**c**) Dimerization curves for EphA2 G131Y in the absence and presence of m-ephrinA1. In this experiment, 212 cells were imaged to obtain 905 data points for the G31Y1 mutant, no ligand, and 419 cells were imaged to obtain 1961 data points for the G131Y mutant in the presence of m-ephrin-A1.

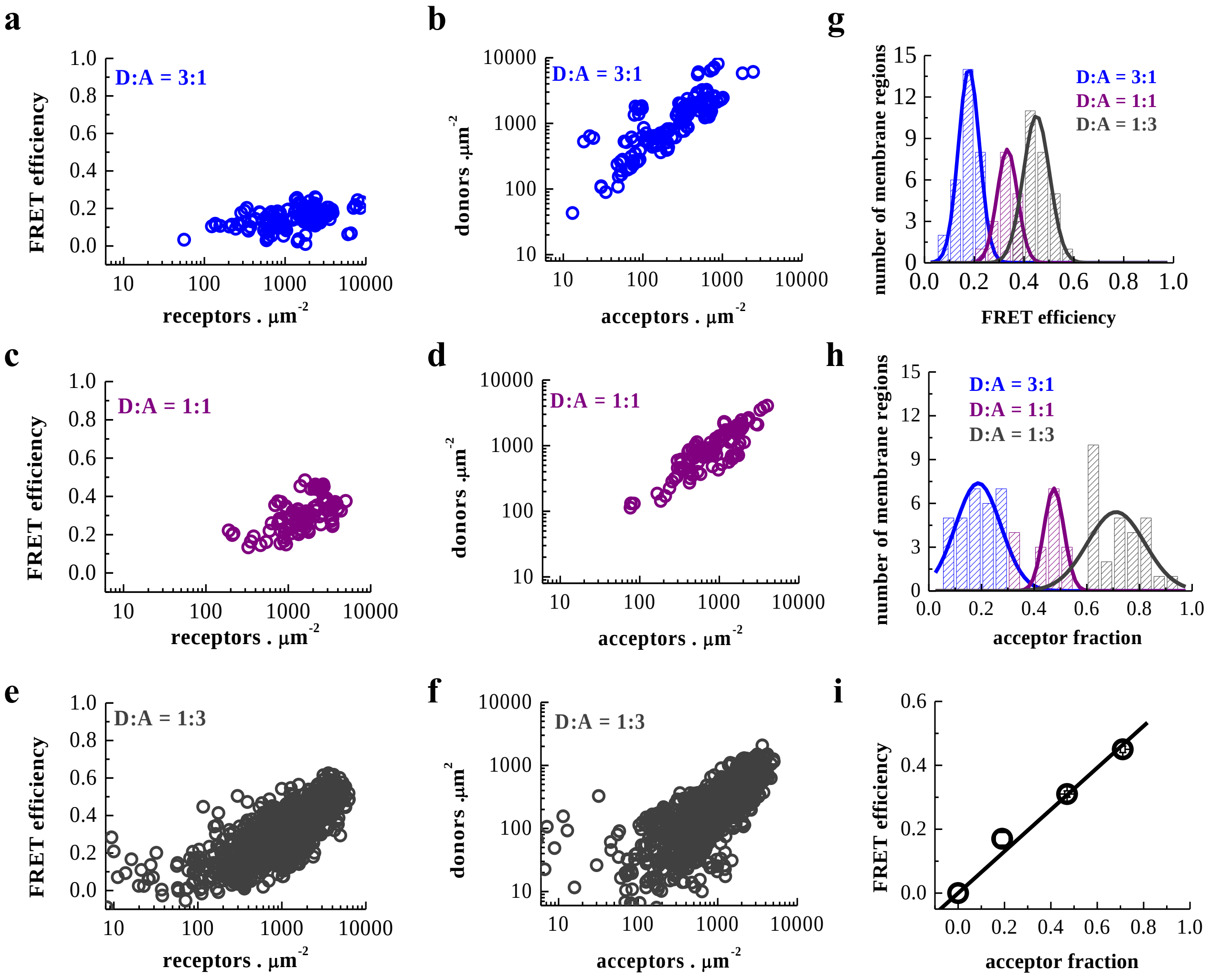

Supplementary Figure 10. The EphA2 R103E mutant forms dimers in response to m-ephrinA1. (a), (c), (e) FRET efficiencies *versus* total receptor concentration when HEK293T cells were co-transfected with DNA encoding EphA2-mTurq R103E (donor) and EphA2-eYFP R103E (acceptor) in 3:1, 1:1, and 1:3 ratios. The cells were stimulated with 200 nM m-ephrin-A1. **(b), (d), (f)** donor concentration *versus* acceptor concentration for the same cells. 50 cells were imaged to obtain 156 data points in (a) and (b), 50 cells were imaged to obtain 122 data points in (c) and (d), and 362 cells were imaged to obtain 1,266 data points in (e) and (f). **(g)** Histograms of the FRET efficiencies in (a), (c), and (e). Only data points above 3,000 receptors per μm^2 were included, because in this case the FRET efficiency depends primarily on the donor-to-acceptor ratio but not on the total concentration. The histograms were fitted with Gaussian functions to obtain the mean FRET efficiencies and the standard errors. (**h)** Histograms of the acceptor fractions in (a), (c), and (e) for total EphA2 concentrations exceeding 3,000 receptors per μm^2, were fitted with Gaussian functions to obtain the mean acceptor fractions and the standard errors. **(i)** The FRET efficiencies are plotted against the acceptor fractions along with the standard errors (smaller than the symbol size). The dependence is linear, indicative of dimer formation[2].

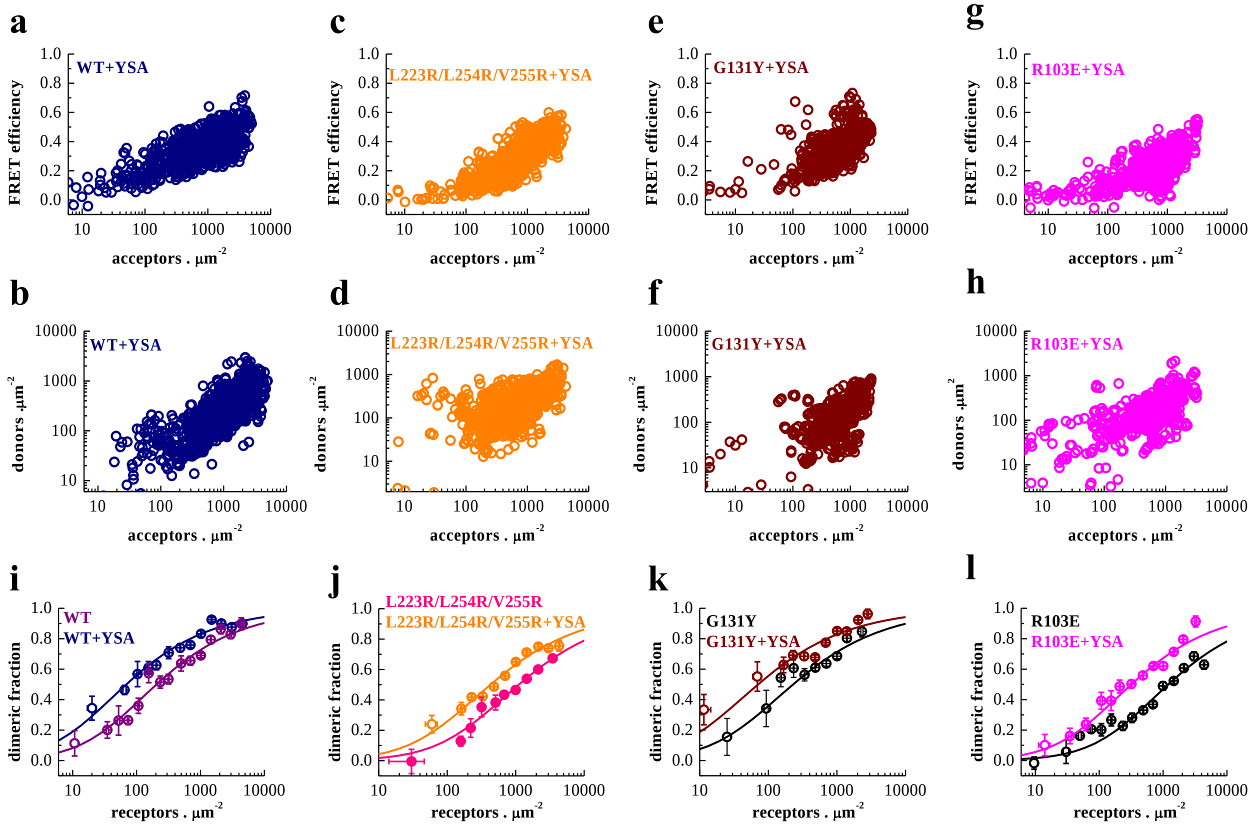

Supplementary Figure 11. **FRET data for EphA2 wild-type and the L223R/L254R/V255R, G131Y and R103E mutants in the presence of YSA peptide ligand.** HEK293T cells were co-transfected with DNA encoding donors and acceptors in a 1:3 ratio and stimulated with 6 µM YSA peptide. (**a**) FRET efficiency versus acceptor concentration for EphA2 wild type. (**b**) Donor concentration versus acceptor concentration for EphA2 wild type. (**c**) FRET efficiency versus acceptor concentration for EphA2 L223R/L254R/V255R. (**d**) Donor concentration versus acceptor concentration for EphA2 L223R/L254R/V255R. (**e**) FRET efficiency versus acceptor concentration for EphA2 G131Y. (**f**) Donor concentration versus acceptor concentration for EphA2 G131Y. (**g**) FRET efficiency versus acceptor concentration for EphA2 R103E. (**h**) Donor concentration versus acceptor concentration for EphA2 R103E. 400 cells were imaged to obtain 1,376 data points in (a) and (b), 269 cells were imaged to obtain 742 data points in (c) and (d), 246 cells were imaged to obtain 699 data points in (e) and (f), and 258 cells were imaged to obtain 748 data points in (g) and (h). (**i**) Comparison of dimerization propensities of EphA2 wild type in the presence and absence of YSA peptide[3]. (**j**) Comparison of dimerization propensities of EphA2 L223R/L254R/V255R in the presence and absence of YSA peptide[3]. (**k**) Comparison of dimerization propensities of EphA2 G131Y in the presence and absence of YSA peptide. (**l**) Comparison of dimerization propensities of EphA2 R103E in the presence and absence of YSA peptide.

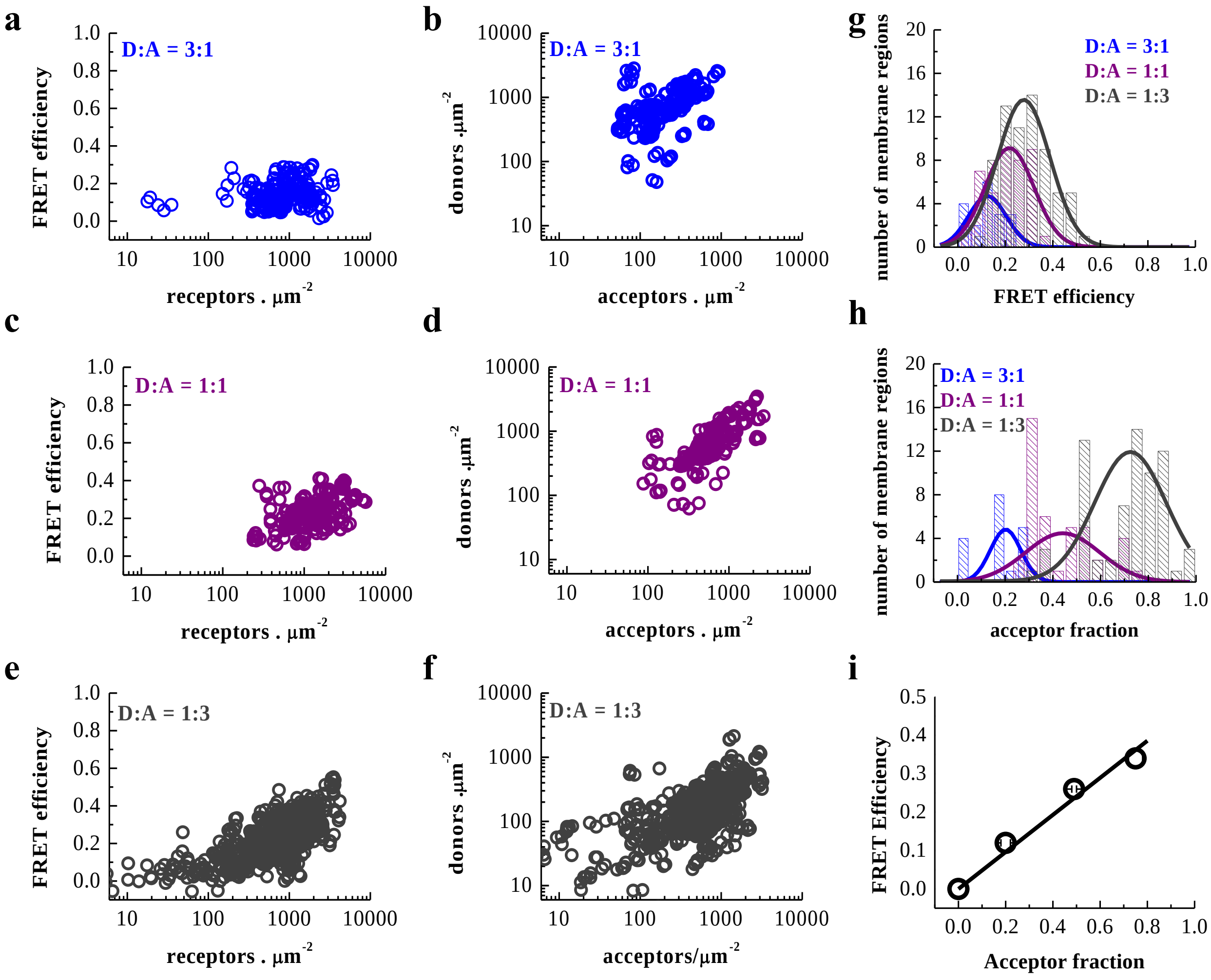

Supplementary Figure 12. The EphA2 R103E mutant forms dimers in response to the YSA peptide ligand. (a), (c), (e) FRET efficiencies *versus* total receptor concentration for HEK293T cells co-transfected with DNA encoding R103E EphA2-mTurq (donor) and R103E EphA2-YFP (acceptor) in 3:1, 1:1, and 1:3 ratios. The cells were stimulated with 6 μM YSA peptide. **(b), (d), (f)** donor concentration *versus* acceptor concentration for the same cells. 50 cells were imaged to obtain 244 data points in (a) and (b), 50 cells were imaged to obtain 198 data points in (c) and (d), and 258 cells to obtain 748 data points in (e) and (f). **(g)** Histograms of the FRET efficiencies in (a), (c), and (e), were fitted with Gaussian functions to obtain the mean FRET efficiencies and the standard errors. Only data points above 3,000 receptors per μm^2 were included, because in this case the FRET efficiency depends primarily on the donor-to-acceptor ratio but not on the total concentration. **(h)** Histograms of the acceptor fractions in (a), (c), and (e) for total concentrations exceeding 3,000 receptors per μm^2, were fitted with Gaussian functions to obtain the mean acceptor fractions and the standard errors. **(i)** The FRET efficiencies are plotted against the acceptor fractions along with the standard errors (which are smaller than the data symbols). The dependence is linear, indicative of dimer formation[2].

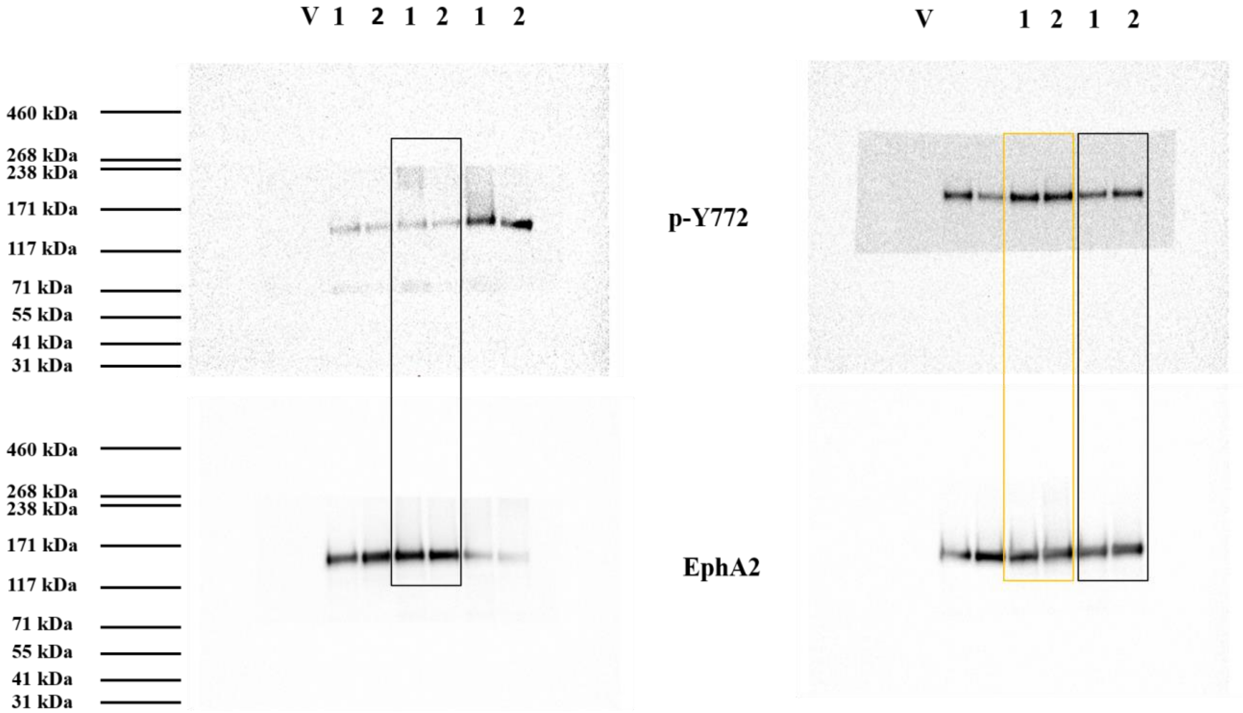

Supplementary Figure 13. Uncropped western blots used to generate the quantifications in **Figure 4e**. Each lane (sample) is obtained from a separate transfection. Membranes were blotted with anti-phospho-Y772 antibody, stripped, and re-blotted with anti-EphA2 antibody. V: vector, 1: WT EphA2 and 2: G131Y mutant. The intensities of the anti-Y722 bands (reporting on tyrosine phosphorylation) and of the anti-EphA2 bands (reporting on expression) were quantified. Matched wild-type and mutant samples with similar receptor expressions were compared (see rectangular boxes). pY772 signals were normalized to the corresponding WT signal in **Figure 4e**. The orange box indicates the samples shown in **Figure 4d**.

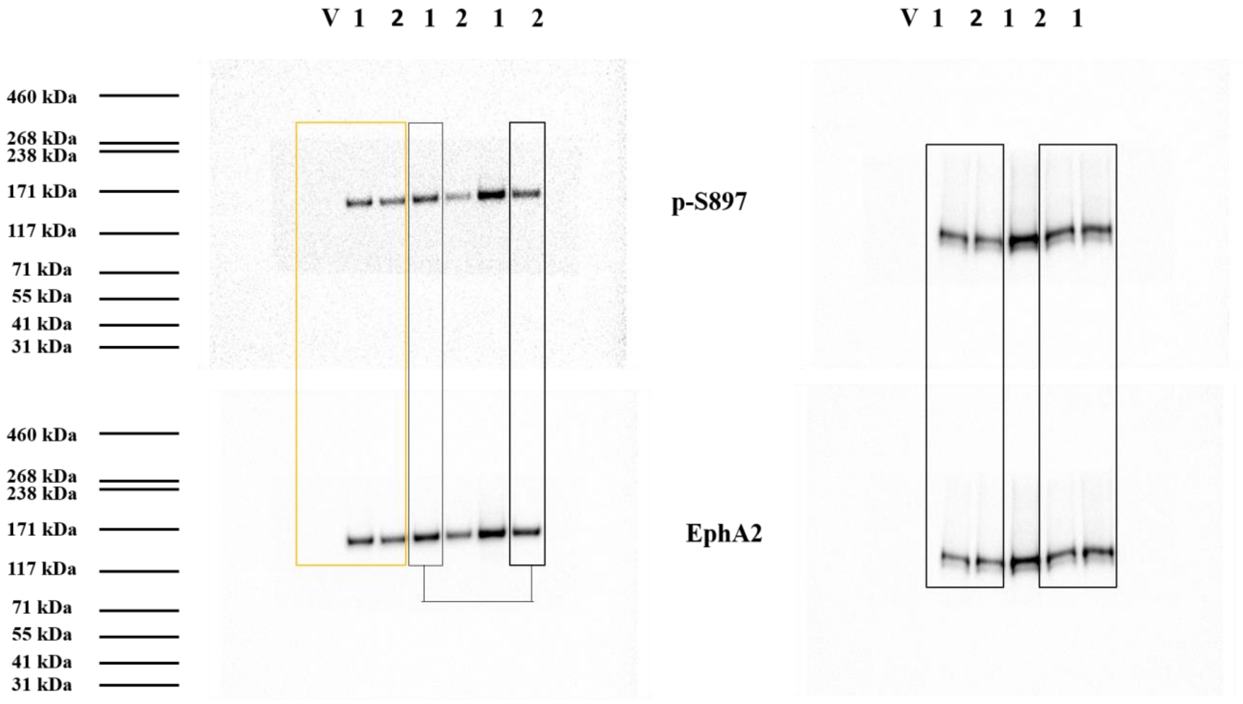

Supplementary Figure 14. Uncropped western blots used to generate the quantifications in **Figure 4g**. Each sample was obtained from a separate transfection. Samples with similar EphA2 expression were directly compared. The four comparisons used for the quantifications in **Figure 4g** are shown in rectangular boxes. The orange box indicates the samples shown in **Figure 4f**. Membranes were blotted with anti-phospho-S897 antibody, stripped, and re-blotted with anti-EphA2 antibody. V: vector, 1: WT EphA2 and 2: G131Y mutant.

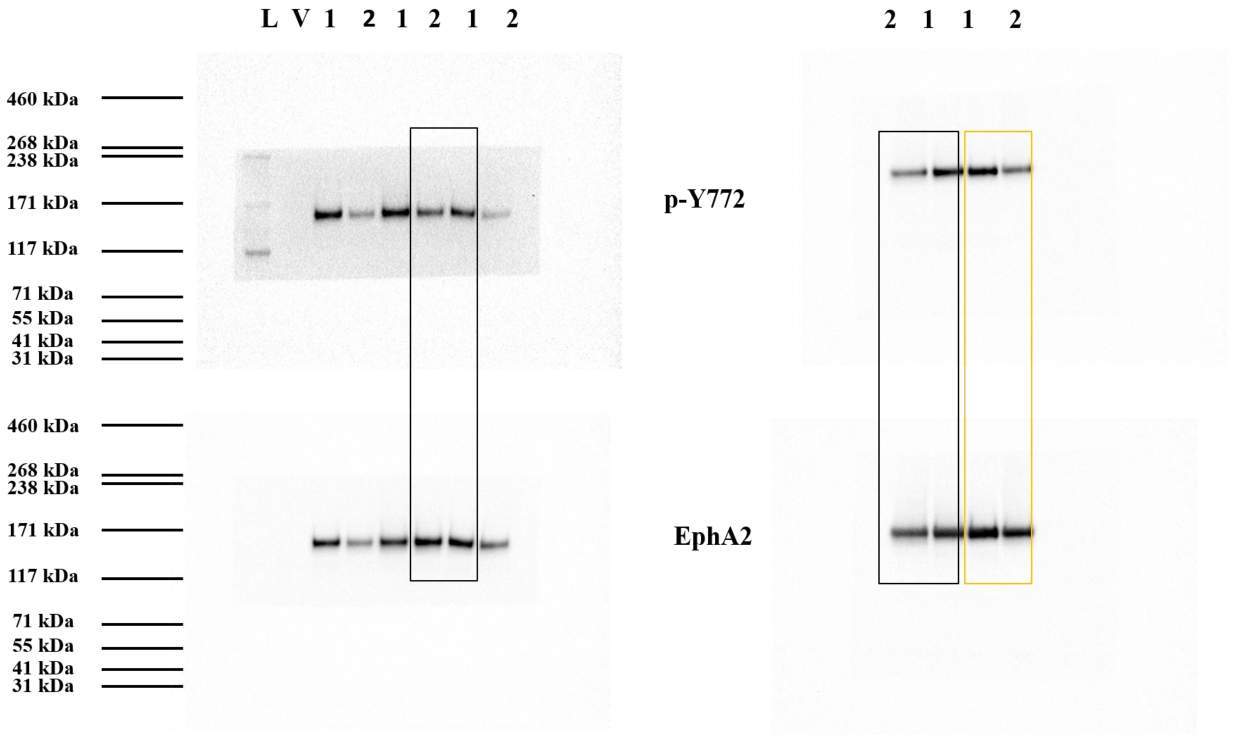

Supplementary Figure 15. Uncropped western blots used to generate the quantifications in **Figure 4i**. Each sample was obtained from a separate transfection. Samples with similar EphA2 expression were directly compared. The three comparisons used for the quantifications in **Figure 4i** are shown in rectangular boxes (note the difference in sample order). The orange box indicates the samples shown in **Figure 4h**. Membranes were blotted with anti-phospho-Y772 antibody, stripped, and re-blotted with anti-EphA2 antibody. V: vector, 1: WT EphA2 and 2: R103E mutant.

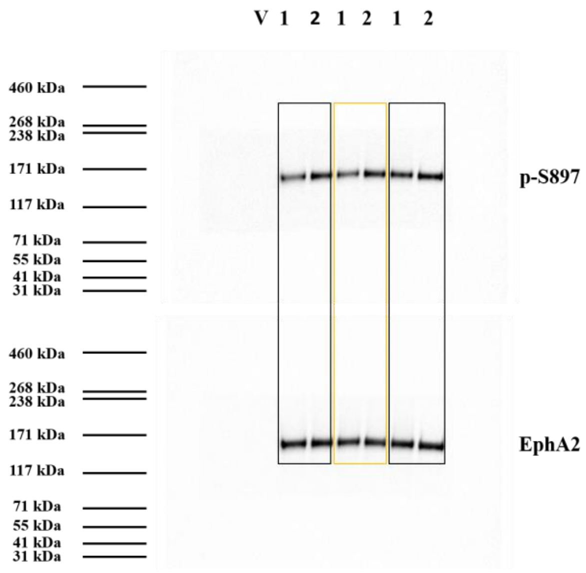

Supplementary Figure 16. Uncropped western blots used to generate the quantifications in **Figure 4k**. Each sample was obtained from a separate transfection. Samples with similar EphA2 expression were directly compared. The three comparisons used for the quantifications in Figure 4k are shown in rectangular boxes. The orange box indicates the samples shown in **Figure 4j**. Membranes were blotted with anti-phospho-S897 antibody, stripped, and re-blotted with anti-EphA2 antibody. V: vector, 1: WT EphA2 and 2: R103E mutant.

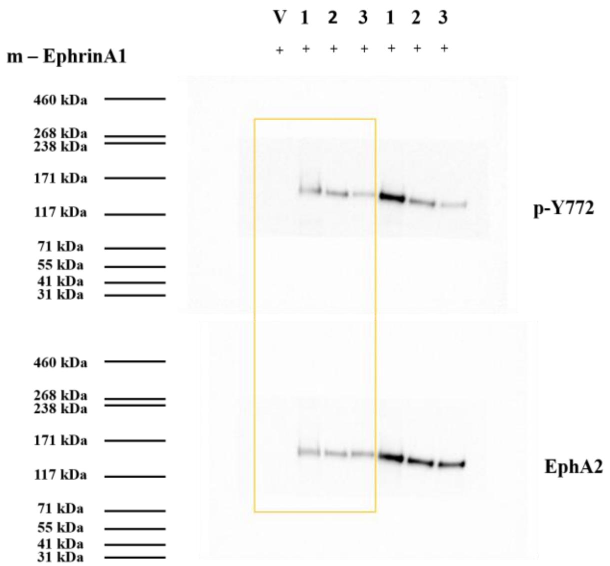

Supplementary Figure 17. Uncropped version of the blot shown in Figure **5d.** The nembrane was blotted with anti-phospho-Tyr772 antibody, stripped, and re-blotted with anti-EphA2 antibody. V: vector, 1: WT EphA2, 2: L223R/L254R/V255R EphA2, and 3: G131Y EphA2.

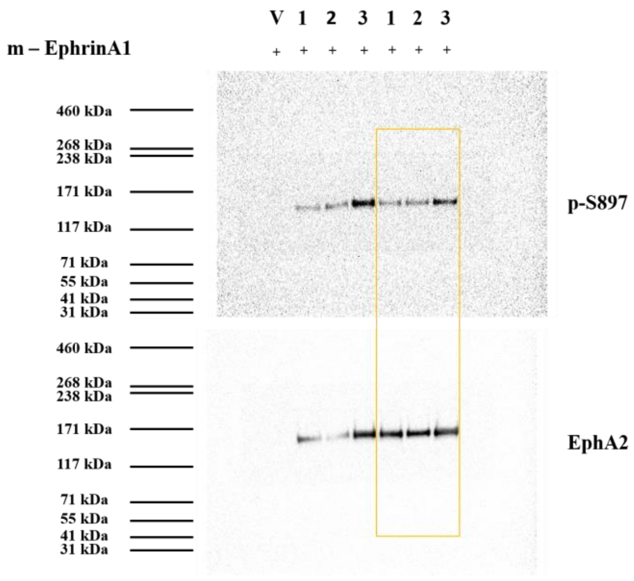

Supplementary Figure 18. Uncropped version of the western blot shown in Figure **5f**. Samples with similar EphA2 expression (orange box) were directly compared. The membrane was blotted with anti-phospho-Ser897 antibody, stripped, and re-blotted with anti-EphA2 antibody. V: vector, 1: WT EphA2, 2: L223R/L254R/V255R EphA2, and 3: G131Y EphA2.

Supplementary References

1 Singh, D. R. *et al.* EphA2 Receptor Unliganded Dimers Suppress EphA2 Pro-tumorigenic Signaling. *Journal of Biological Chemistry* **290**, 27271-27279 (2015).
2 Veatch, W. & Stryer, L. The dimeric nature of the gramicidin A transmembrane channel: conductance and fluorescence energy transfer studies of hybrid channels. *J Mol Biol* **113**, 89-102 (1977).
3 Singh, D. R., Pasquale, E. B. & Hristova, K. A small peptide promotes EphA2 kinase-dependent signaling by stabilizing EphA2 dimers. *Biochim Biophys Acta* **1860**, 1922-1928.
